# In Silico Identification of Molecular Interactions of the Emerging Contaminant Octyl Methoxycinnamate (OMC) on HPT Axis: Implications for Humans and Zebrafish

**DOI:** 10.3390/ph18121897

**Published:** 2025-12-16

**Authors:** Margarida Lorigo, Luiza Breitenfeld, Marta S. Monteiro, Amadeu M. V. M. Soares, Carla Quintaneiro, Elisa Cairrao

**Affiliations:** 1 FCS-UBI, Faculty of Health Sciences, University of Beira Interior, 6200-506 Covilhã, Portugal; margarida.lorigo@gmail.com (M.L.); luiza@fcsaude.ubi.pt (L.B.); 2 RISE-Health, Department of Medical Sciences, Faculty of Health Sciences, University of Beira Interior, 6200-506 Covilhã, Portugal; 3 Department of Biology & CESAM (Centre for Environmental and Marine Studies), University of Aveiro, 3810-193 Aveiro, Portugal; mmonteiro@ua.pt (M.S.M.); asoares@ua.pt (A.M.V.M.S.); cquintaneiro@ua.pt (C.Q.)

**Keywords:** thyroid disruption, T3 hormone, propylthiouracil, Lipinski’s rule-of-five, ADMET properties, molecular docking

## Abstract

**Background/Objectives:** Thyroid hormones (THs) regulate almost all physiological processes in vertebrates via specific mechanisms exercised spatiotemporally throughout the lifespan. The TH signalling can be impaired by thyroid-disrupting chemicals (TDCs) capable of disrupting the hypothalamic–pituitary–thyroid (HPT) axis. Octyl methoxycinnamate (OMC) (also designated octinoxate), one of the most widely used ultraviolet (UV) filters, has emerged as an environmental contaminant and has raised significant concerns recently due to its disruptive effects as TDC on humans and animals. Although the disruption of TH homeostasis has been reported, its exact modes of action (MoA) remain largely unknown. Our study aimed to provide a comparative information on the molecular interactions of OMC on TH signalling in humans and zebrafish. **Methods**: In silico approaches were performed comparing OMC with endogenous thyroid hormone T3 and the anti-thyroid drug propylthiouracil (PTU). **Results**: Our findings suggested a key role of OMC on the corticotrophin-releasing hormone receptor (crhr2), thyrotropin receptor (TSHR/tshr), and thyroid nuclear receptors (TR/tr-α and -β). At the hypothalamic level, a favourable binding of OMC to zebrafish crhr2 was found, involving ALA86, CYS44, HIS89, ILE63, ILE64, LEU92, PRO87, PRO88, SER48, and THR47. At the pituitary level, OMC was bound to human TSHR by the amino acid residues ASN590, GLU506, ILE583, ILE640, LEU570, MET572, PRO571, SER505, TYR667, VAL502, VAL586, ALA644, LEU587, MET637, SER641, and TYR582 and to zebrafish tsrh by ASN589, ILE639, MET636, ILE582, LEU569, LEU586, VAL501, and VAL585. Concerning nuclear receptors, OMC showed a more favourable binding energy of T3, involving the shared residues PHE218 and MET259 with T3 in both species. For human TRβ, OMC shared T3 with residues ILE 275, ILE276, LEU346, PHE269, PHE272, THR273, ALA279, ASN331, HIS435, LEU330, MET310, MET313, and PHE455. No similar residues were obtained for zebrafish trβ compared with the humans. **Conclusions**: Overall, the action of OMC seems to agree with primary hypothyroidism (anti-thyroid action) mimicking the T3 hormone. This investigation demonstrates that OMC acts as a potential TDC and provides new insights into its disruptive action on the HPT axis.

## 1. Introduction

Thyroid hormones (THs) regulate practically all physiological processes in vertebrates through specific actions exercised spatiotemporally throughout life and are mediated by both systemic and local regulation [1]. Notably, TH signalling can be impaired by thyroid-disrupting chemicals (TDCs), which are capable of interfering with the hypothalamic–pituitary–thyroid (HPT) axis at multiple levels, including TH synthesis, transport, binding, absorption, and local tissue metabolism [2,3,4].

In silico approaches are useful tools for identifying potential TDCs. Molecular docking, in particular, is a valuable tool for studying ligand–protein binding interactions and can be applied to identify molecular initiating events (MIEs) within the adverse outcome pathway (AOP) framework [5,6]. Understanding the complex biology of toxicological effects in vivo by identifying key events (KEs) in TH-mediated pathways will contribute to safeguarding human and ecological health from TDC exposure. The high degree of conservation across species provides a unique opportunity to apply new approach methodologies (NAMs) more broadly in risk/hazard assessments [7]. In this context, developing zebrafish embryos is a promising model for evaluating the effects of HPT axis disruption by TDCs [8]. The zebrafish genome has been fully sequenced and has approximately 70% homology with human genes, including 84% of genes associated with human diseases [9]. These observations support the relevance of extrapolating thyroid toxicity effects from zebrafish to humans [10,11,12].

Ultraviolet (UV) filters have emerged as an important class of TDCs. Due to increasing consumer awareness about the harmful effects of UV exposure, the use of personal care products (PCPs) containing UV filters has increased substantially [13]. Octyl methoxycinnamate (OMC), one of the most-used UV filters, has been identified as an environmental contaminant and has raised significant concern in recent years due to its disruptive effects on both humans and animals [14,15,16,17,18]. Although the disruption of TH homeostasis has already been reported in humans [19,20,21] and zebrafish [22,23,24], the exact mechanisms underlying these effects remain largely unknown. Several studies have attempted to clarify the toxicity of OMC to draw more definitive conclusions regarding its long-term effects on human health. At the environmental level, OMC (in ng/L) was detected in seawater from four Italian beaches [25], as well as in rivers and lakes in Switzerland [26,27]. Moreover, OMC was detected in swimming pool water (in μg/L) [27], as well as in tap and drinking water samples (in ng/L) [28], suggesting that conventional water treatment processes are largely ineffective at fully removing this contaminant [26,28]. On the other hand, the presence of OMC in various commercially important fish species [26], suggests that its bioaccumulation may pose risks to both wildlife and humans through dietary exposure via the food chain [29]. OMC can readily penetrate the human skin and was detected in human plasma (0.016 μg/mL), urine (0.006 μg/mL) [30], breast milk (detected in 60% of samples [31]), and breast tissue (detected in 74% of samples; range 0–58.7 ng/g tissue [32]). Furthermore, a recent study has identified OMC as an obesogenic EDC [33], suggesting a potential role in obesity-related alterations in epigenetic programming. In addition, OMC bioaccumulation has been reported in aquatic organisms [26,27,34], and maternofoetal transfer has been observed in other mammals [35]. Taken together, these findings underscore the urgent need for the comprehensive assessment of the toxicity of OMC regarding both human and environmental health.

This study aimed to investigate the molecular interactions of the environmental contaminant OMC on TH signalling pathways. The mode of interaction of OMC with molecular targets of the HPT axis was analysed and compared with that of endogenous thyroid hormone 3,3′,5-triiodothyronine (T3) and anti-thyroid drug propylthiouracil (PTU) (Figure S1). T3 and PTU were selected as reference substances, as T3 enhances TH signalling whereas PTU interferes with TH production by inhibiting thyroid peroxidase in zebrafish and inhibiting iodothyronine deiodinases in mammals [36]. Both substances are well-established model substances in thyroid disruption studies [37,38] and are widely used for the experimental induction of hyperthyroidism [39,40] and hypothyroidism by in vitro studies [40,41,42], respectively. To assess potential alterations in TH-signalling, proteins involved in different cascade events of the HPT axis, including thyroid stimulation, thyroid-related receptors, and TH transport, were selected for analysis. The pharmacokinetic properties of all three compounds were evaluated, and in silico molecular docking studies were performed. Analyses were conducted for both *Homo sapiens* and *Danio rerio* to enable cross-species comparisons and to assess the relevance of findings for human health.

## 2. Results

### 2.1. Molecular Docking

In silico simulations by molecular docking were performed to unveil the modes of interaction of T3, PTU, and OMC with the HPT axis. All simulations were performed considering a semi-rigid docking (protein as rigid and ligand as flexible). Results can be seen in Table 1, Figure 1, Figure 2, Figure 3, Figure 4, Figure 5 and Figure 6, and Figures S2–S7.

#### 2.1.1. Thyrotropin-Releasing Hormone Receptor

**Figure 1:**
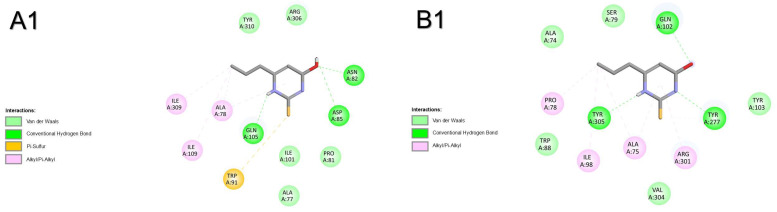

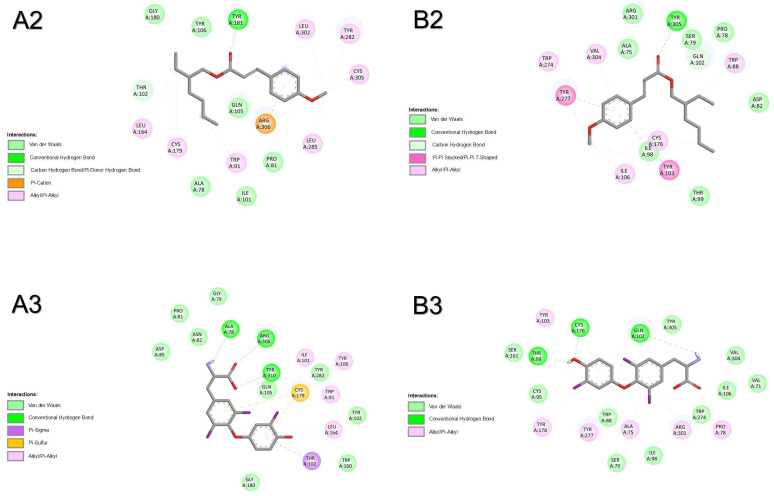
Atomic interactions by Discovery Studio of the complex between the ligands, (**1**) propylthiouracil (PTU), (**2**) octyl methoxycinnamate (OMC), and (**3**) triiodothyronine (T3) with thyrotropin-releasing hormone receptor from (**A**) humans and (**B**) zebrafish.

Results from molecular docking with the thyrotropin-releasing hormone receptor of both species show that T3 had the most favourable binding energy, followed by OMC and PTU (Table 1). For human receptor TRHR (Figure 1A and Figure S13), the shared binding of compounds only involved the amino acid residue GLN105. The binding of OMC was also shared with T3 by amino acid residues ARG306, CYS179, LEU164, THR102, TYR181, and TYR282. One H-bridge with ALA78 (2.454 Å) was involved in the binding of T3 to TRHR. For the zebrafish receptor (trhr, Figure 1B and Figure S13), compounds shared amino acid residues GLN102, TYR277, and TYR301 to bind to the active centre of the receptor. The binding of OMC was also shared with T3 by amino acid residues CYS176, ILE106, and TYR103. No H-bridges were involved in the binding of the three compounds to trhr. The most prevalent atomic interactions observed for TSHR/tshr were Van der Walls, followed by π-alkyl/alkyl and conventional H-Bond (Figure S14).

#### 2.1.2. Corticotropin-Releasing Hormone Receptor 2

**Figure 2:**
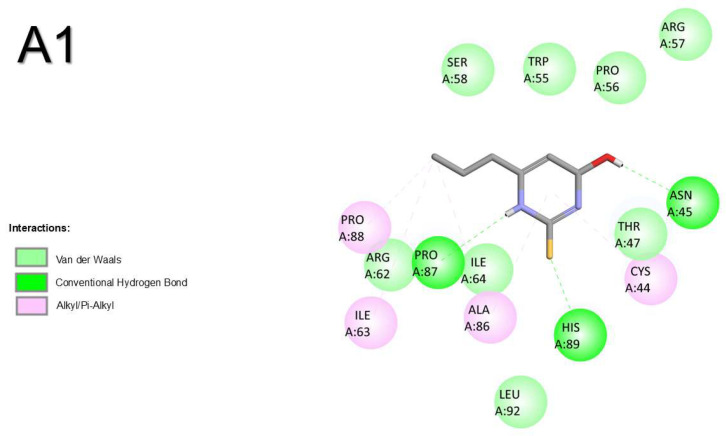

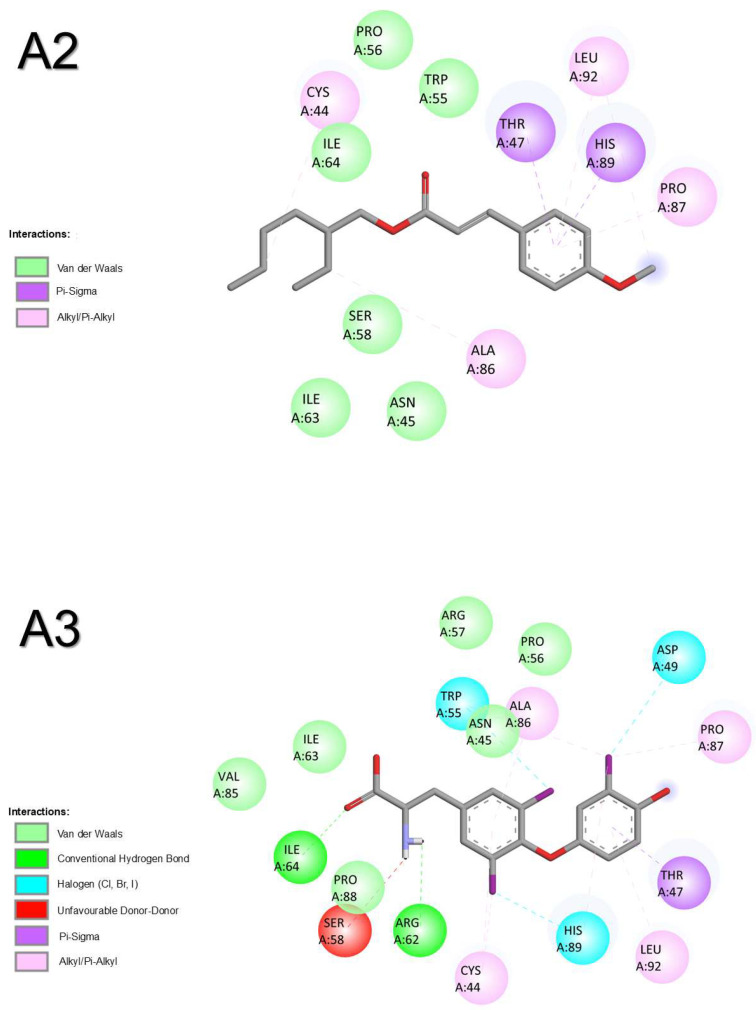
Atomic interactions by Discovery Studio of the complex between the ligands, (**1**) propylthiouracil (PTU), (**2**) octyl methoxycinnamate (OMC), and (**3**) triiodothyronine (T3) with corticotropin-releasing hormone receptor 2 from (**A**) zebrafish.

Molecular docking with corticotropin-releasing hormone receptor 2 (crhr2) was only performed for zebrafish. The results show that OMC had the most favourable binding energy, followed by PTU and T3 (Table 1). The binding of all compounds was shared by amino acid residues SER48, THR47, ALA86, HIS89, PRO87, PRO88, and ILE63. The binding of OMC was also shared with T3 by amino acid residues CYS44, LEU92, and ILE64 (Figure 2). No H-bridges were involved in the binding of the three compounds to crhr2. The most prevalent atomic interactions observed for crhr2 were Van der Walls, followed by alkyl/π-alkyl and conventional H-bond (Figure S15).

#### 2.1.3. Thyroid-Stimulating Hormone Receptor

**Figure 3:**
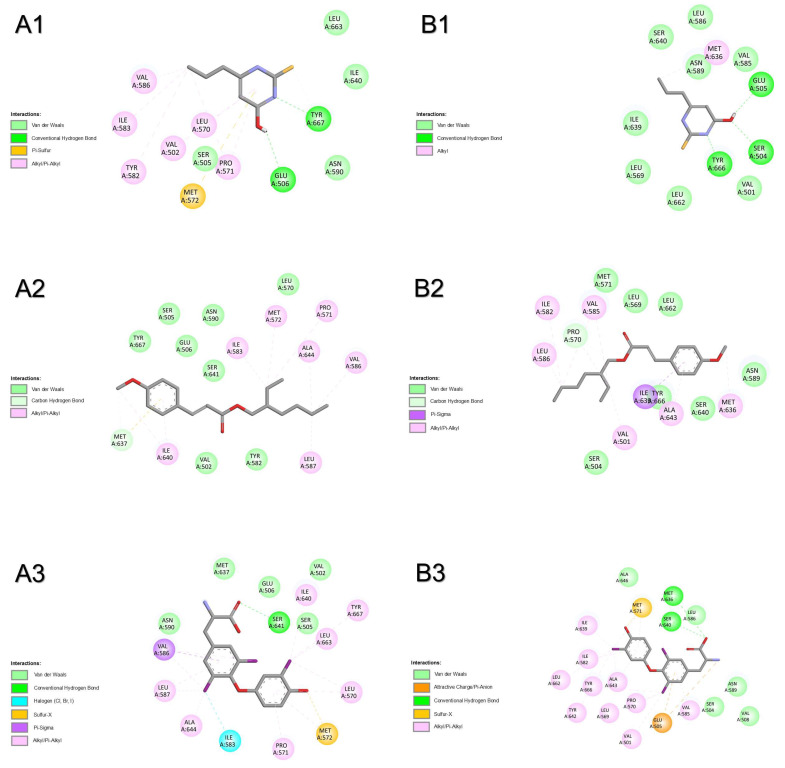
Atomic interactions by Discovery Studio of the complex between the ligands, (**1**) propylthiouracil (PTU), (**2**) octyl methoxycinnamate (OMC), and (**3**) triiodothyronine (T3) with thyroid-stimulating hormone receptor (or thyrotropin receptor) from (**A**) humans and (**B**) zebrafish.

Results from molecular docking with thyroid-stimulating hormone receptor of both species show that OMC had the most favourable binding energy, followed by PTU and T3 in humans or the inverse in zebrafish (Table 1). For human receptor TSHR (Figure 3A), the binding of compounds was shared by almost all amino acid residues: ASN590, GLU506, ILE583, ILE640, LEU570, MET572, PRO571, SER505, TYR667, VAL502, and VAL586. The binding of OMC was also shared with T3 by amino acid residues ALA644, LEU587, MET637, and SER641, and with PTU by TYR582. One H-bridge with GLN489 (2.631 Å) was involved in the binding of T3 to TSHR. For zebrafish receptor tshr (Figure 3B and Figure S16), compounds shared the binding by amino acid residues ASN589, ILE639, and MET636. The binding of OMC was also shared with T3 by amino acid residues ILE582, LEU569, LEU586, VAL501, and VAL585. No H-bridges were involved in the binding of the three compounds to tshr. The most prevalent atomic interactions observed for tshr were alkyl, followed by Van der Walls (Figure S17).

#### 2.1.4. Transthyretin

**Figure 4:**
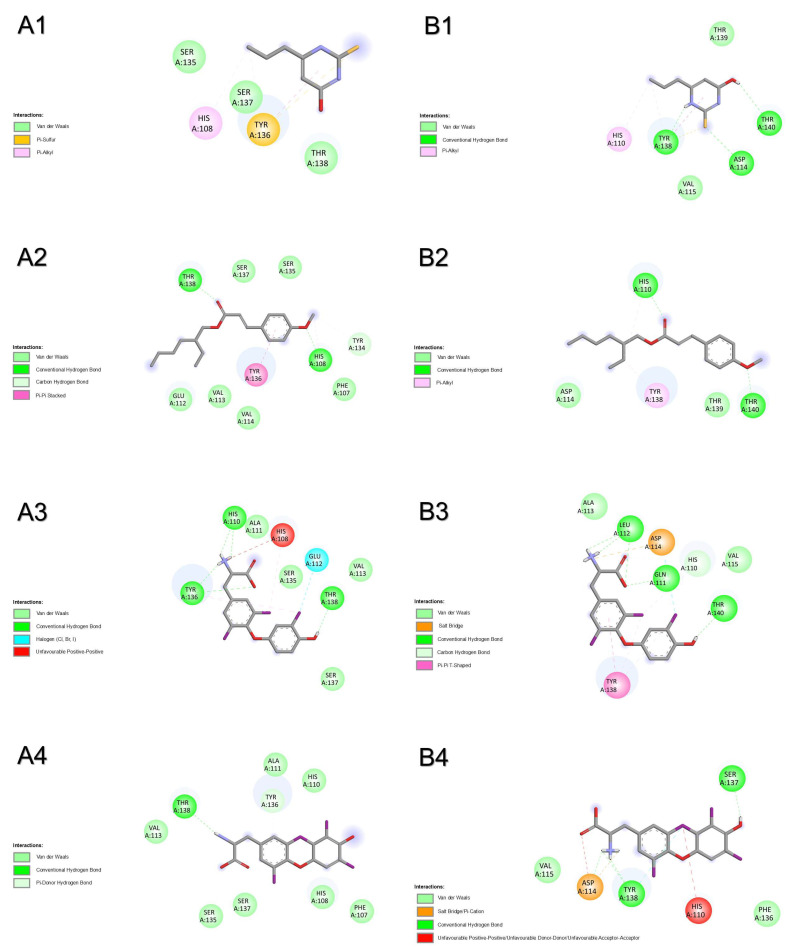
Atomic interactions by Discovery Studio of the complex between the ligands, (**1**) propylthiouracil (PTU), (**2**) octyl methoxycinnamate (OMC), (**3**) triiodothyronine (T3), and (**4**) tetraiodothyronine (T4) with transthyretin from (**A**) humans and (**B**) zebrafish.

Results from molecular docking with transthyretin of both species show that T3 had the most favourable binding energy, followed by T4, OMC, and PTU (Table 1). For human transport protein TTR (Figure 4A and Figure S18), the binding of compounds was shared by amino acid residues HIS108, THR138, and TYR136. The binding of OMC was also shared with T3 by the amino acid residue GLU112. No H-bridges were involved in the binding of the four compounds to TTR. For zebrafish transport protein ttr (Figure 4B and Figure S18), the binding of compounds was shared by almost all amino acid residues: ASP114, HIS110, THR140, and TYR138. Some H-bridges were involved in the binding of PTU (ALA130, 2.270 Å) and T3 (LEU132, 2.426 Å and SER137, 2.076 Å) to ttr. The most prevalent atomic interactions observed for TTR were Van der Walls (Figure S19A). For ttr, the most prevalent atomic interactions observed were conventional H-bond followed by Van der Walls (Figure S19B).

#### 2.1.5. Thyroid Hormone Receptor Alpha

**Figure 5:**
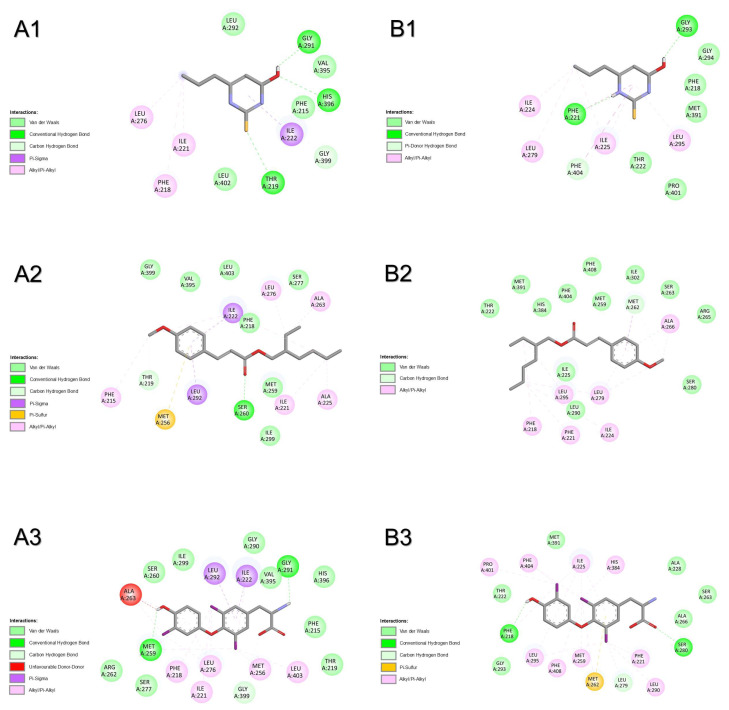
Atomic interactions by Discovery Studio of the complex between the ligands, (**1**) propylthiouracil (PTU), (**2**) octyl methoxycinnamate (OMC), and (**3**) triiodothyronine (T3) with thyroid hormone receptor alpha from (**A**) humans and (**B**) zebrafish.

Results from molecular docking with thyroid hormone receptor alpha of both species show that OMC had the most favourable binding energy, followed by PTU and T3 in humans or the inverse in zebrafish (Table 1). For human nuclear receptor TRα (Figure 5A and Figure S20), the binding of compounds was shared by amino acid residues ILE222, LEU292, PHE215, and PHE218. The binding of OMC was also shared with T3 by amino acid residues ALA263, ILE221, LEU276, MET256, MET259, SER260, and SER277. One H-bridge with ILE378 (2.203 Å) was involved in the binding of T3 to TRα. For zebrafish nuclear receptor trα (Figure 5B and Figure S20), the binding of compounds was shared by amino acid residues ILE225, PHE218, PHE221, and PHE404. The binding of OMC was also shared with T3 by amino acid residues ALA266, HIS384, LEU279, MET259, MET262, and SER280, and with PTU by ILE224. No H-bridges were involved in the binding of the three compounds to trα. The most prevalent atomic interactions observed for TRα were Van der Walls, followed by π-alkyl/alkyl and conventional H-bond (Figure S21A). For trα, the most prevalent atomic interactions observed were Van der Walls, followed by π-alkyl/alkyl (Figure S21B).

#### 2.1.6. Thyroid Hormone Receptor Beta

**Figure 6:**
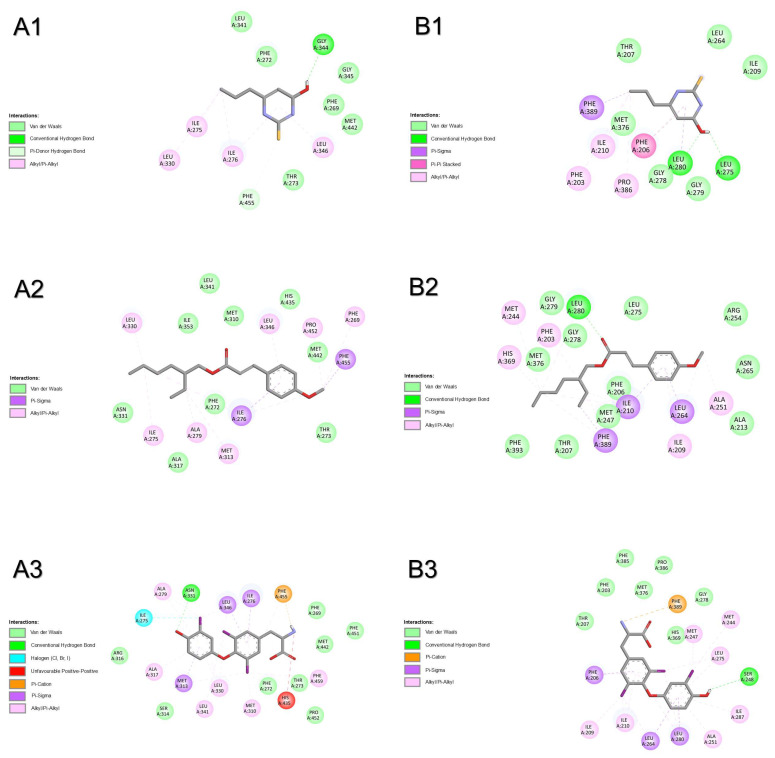
Atomic interactions by Discovery Studio of the complex between the ligands, (**1**) propylthiouracil (PTU), (**2**) octyl methoxycinnamate (OMC), and (**3**) triiodothyronine (T3) with thyroid hormone receptor beta from (**A**) humans and (**B**) zebrafish.

Results from molecular docking with thyroid hormone receptor beta show that OMC had the most favourable binding energy, followed by T3 and PTU (Table 1). For human nuclear receptor TRβ (Figure 6A and Figure S22), the binding of compounds was shared by amino acid residues ILE 275, ILE276, LEU346, PHE269, PHE272, and THR273. The binding of OMC and T3 was also shared by amino acid residues ALA279, ASN331, HIS435, LEU330, MET310, MET313, and PHE455. One H-bridge was involved in the binding of PTU (THR329, 2.323 Å) to TRβ. For zebrafish nuclear receptor trβ (Figure 6B and Figure S22), the binding of compounds was shared by almost all amino acid residues: ILE210, LEU264, LEU275, LEU280, MET376, PHE203, PHE206, PHE389, and THR207. One H-bridge was involved in the binding of PTU (ASN265, 2.027 Å) and T3 (MET247, 1.872 Å) to trβ. The most prevalent atomic interactions observed for TRβ were π-alkyl/alkyl, followed by Van der Walls (Figure S23A). For trβ, the most prevalent atomic interactions observed were π-alkyl/alkyl, followed by Van der Walls, π-sigma, and conventional H-bond (Figure S23B).

## 3. Discussion

In humans, the hypothalamus secretes TRH, which stimulates the production and release of thyrotropin (TSHβ) by the pituitary. TSHβ then acts on the thyroid, stimulating the synthesis and release of TH by the thyroid follicles [43]. These human physiological mechanisms constitute the HPT axis, where TRH serves as the primary stimulator of TSH secretion [44]. In non-mammalian vertebrates, this role appears to be shared by crh alongside trh. crh exerts a double hypophysiotropic action (in the pituitary) on both the HPT axis and the hypothalamus–pituitary–inter-renal (HPI) axis [43], the latter being the functional equivalent of the hypothalamic–pituitary–adrenal (HPA) axis in humans. In these cases, crh acts as a common regulator of the two endocrine axes, by binding to two types of receptors (crhr1 and crhr2), and also plays a role in modulating the cardiovascular system [45,46]. The secretion of tsh induced by crh is mediated by crhr2 in the thyrotrophs, while the secretion of acth (adrenocorticotrophin) is mediated by crhr1 in the corticotrophs. Therefore, crh exerts a stimulatory effect on the release of tsh and acth, influencing the activity of both endocrine axes. Consequently, crh and trh, along with tshβ, play essential roles in regulating the HPT axis. To our knowledge, in zebrafish, it remains unclear which of the two hypothalamic hormones is more important for regulating TH levels, but the transcription levels of both have already been used in the assessment of OMC-induced endocrine disruption [22,23]. Evidence from animal models and cell line studies suggests that all three hormones are subject to TH-mediated feedback regulation mechanisms, either at the hypothalamic level [47], the pituitary level [48,49], or both, in order to maintain physiological homeostasis. In this context, the crosstalk between these endocrine axes constitutes a potential MoA for TDCs that should be analysed simultaneously to better clarify endocrine differences between the two species.

The TRHR simulations show that the binding to OMC was favourable and mainly shared with T3 by amino acid residues ARG306, CYS179, LEU164, THR102, TYR181, and TYR282. Similar results were attained for zebrafish trhr, where OMC shared with T3 amino acid residues GLN102, TYR277, TYR301, CYS176, ILE106, and TYR103. To our knowledge, there is no information in the literature about TDCs binding to TRHR. However, the same amino acid residues (ARG306, CYS179, LEU164, TYR181, and TYR282) were reported in the binding of TRH (natural ligand) to TRHR [50], which is suggestive that OMC competes with TSH to binding TSHR.

Further, to address the role of crhr in the zebrafish HPT axis, simulations with crhr2 were also performed. The results show that OMC had the most favourable binding energy of the three compounds. These findings are very important since they prove the role of OMC as TDC, with an action in crhr2 to regulate the zebrafish HPT axis, agreeing with the more prominent physiological effects observed in this specie. Considering these simulations (TRHR/trhr and crhr2), OMC seems to modulate the hypothalamic control. To our knowledge, only one recent article analysed the effect of a TDC (ethiprole, insecticide) and its two main metabolites (ethiprole sulfone (M1) and ethiprole sulfide (M2)) on human CRH binding. The authors reported a favourable binding of M1 to human CRHR, similar to that of the positive control (antagonist CP-376395). However, we cannot make a direct comparison with these results, despite their similarity, since the authors carried out their studies for CRHR1 and not CRHR2 (a receptor linked to the zebrafish HPT axis) and considered an *H. Sapiens* specie [51]. However, our findings agree with the in vitro studies in zebrafish larvae, where exposure to OMC induced the up-regulation of *crh* [22] (30 µmol/L) and *trh* [23] (>3 µmol/L). In this sense, more studies are urgently needed to disclosure the crh receptors (crhr1 and crhr2)’s role in the zebrafish HPT axis modulation, crucial to infer the physiopathological implications of TDCs in humans.

In human physiological situations, TRH will stimulate the pituitary gland to produce and secrete TSH [43]. Our computational simulations suggest that OMC had the most favourable binding energy (TSHR: ∆G = −5.68 kcal/mol, k_i_ = 68.23 µmol/L, tshr: ∆G = −5.86 kcal/mol, k_i_ = 50.89 µmol/L). For human TSHR, the binding of compounds was shared by almost all amino acid residues: ASN590, GLU506, ILE583, ILE640, LEU570, MET572, PRO571, SER505, TYR667, VAL502, and VAL586. The binding of OMC was also shared with T3 by amino acid residues ALA644, LEU587, MET637, and SER641, and with PTU by TYR582. For zebrafish tshr, compounds shared the binding by amino acid residues ASN589, ILE639, and MET636. The binding of OMC was also shared with T3 by amino acid residues ILE582, LEU569, LEU586, VAL501, and VAL585. To our knowledge, no studies have been conducted on this receptor with T3, PTU, or OMC. However, a recent study by Xu, X., et al. [52] reported that the halogenated benzoylurea pesticide flufenoxuron (a TDC) binds to the extracellular domain of TSHR, involving the same amino acids observed for OMC binding. In summary, the binding of OMC to TSHR/tshr appears to be one of the main MoA for this TDC. Indeed, exposure to OMC induces the up-regulation of *tshβ* [23] at a concentration of 30 µmol/L, and *tshr* [22,23] at concentrations of up to 10 µmol/L in zebrafish larvae.

After synthesis, THs are released into the bloodstream, where they act on target cells. Their distribution/transport is largely mediated by binding proteins, in which TTR is the primary carrier of TH in fish [53,54] and is, therefore, a key target of endocrine disruption, which can alter circulating TH levels. OMC is a lipophilic molecule (https://pubchem.ncbi.nlm.nih.gov/compound/5355130, accessed on 22 September 2025), so it is expected to circulate in the blood bound to plasma proteins, similar to TH, while also having a greater ability to cross the membrane plasma. According to Lipinski’s rule-of-five, OMC meets only one criterion, suggesting it may face some limitations in membrane absorption/permeation. Despite that, OMC appears to cross cell membranes more effectively than T3, which meets two of Lipinski’s criteria and therefore may have even more restricted permeability. According to the literature, exposure to 10 µmol/L of OMC leads to the down-regulation of the *ttr* gene in zebrafish larvae, along with decreased levels of T3 and T4 at OMC concentrations of up to 3 and 1 µmol/L, respectively [22]. Although Ka, Y. and K. Ji [23] did not evaluate *ttr* gene expression, they also reported reduced levels of TH following the exposure of zebrafish larvae to 30 µmol/L of OMC. Reduced *ttr* expression levels lead to a higher proportion of TH circulating in their free form, making them more susceptible to metabolism and clearance from the body, ultimately leading to lower TH levels [55]. In this study, T3 and T4 presented the strongest binding to the active TTR-binding centre, as expected. OMC also showed a favourable binding to this site (TTR: ∆G = −3.66 kcal/mol, k_i_ = 2090.00 µmol/L; ttr: ∆G = −3.53 kcal/mol, k_i_ = 2600.00 µmol/L). However, it must be noted that the k_i_ obtained were extremely high, suggesting that these bonds are likely weak and easily broken, and that, on the other hand, very high concentrations of OMC may be necessary for significant binding. In this study, residues HIS108, THR138, and TYR136 were identified in the binding of all compounds to human TTR. For zebrafish ttr, the binding involved ASP114, HIS110, THR140, and TYR138. To our knowledge, no studies have investigated the binding activity of T3 or PTU with transthyretin. Similarly, there is no scientific evidence supporting shared binding sites between OMC and T4. These findings suggest that the binding of OMC to transthyretin may not be the main MoA by which OMC induces endocrine disruption. In fact, only two studies have evaluated the binding interaction between OMC and TTR. Cotrina, E. Y., et al. [13] demonstrated that OMC did not interfere with TTR/125I-T4 binding. However, pre-incubation of the recombinant TTR with OMC increased resistance to urea-induced denaturation compared with recombinant TTR alone. Moreover, another study by Cahova, J., et al. [56] suggests that OMC does not behave as a TTR ligand. Together, these findings support our hypothesis.

After distribution/transport, THs reach target cells, where they exert their biological effects, both genomic and non-genomic. The genomic actions of T3 are mediated by its binding to nuclear receptors, namely TRα and TRβ [57,58]. In this study, all compounds exhibited favourable binding affinities for both TRα and TRβ. Interestingly, OMC showed the strongest binding energy in the different simulations (TRα: ∆G = −6.92 kcal/mol, k_i_ = 8.49 µmol/L; trα: ∆G = −7.83 kcal/mol, k_i_ = 1.83 µmol/L; TRβ: ∆G = −7.88 kcal/mol, k_i_ = 1.68 µmol/L; trβ: ∆G = −8.13 kcal/mol, k_i_ = 1.09 µmol/L). Regarding human TRα, all compounds showed binding through key residues ILE222, LEU292, PHE215, and PHE218 at the active centre of the receptor. Notably, OMC shared binding residues with T3, including amino acid residues ALA263, ILE221, LEU276, MET256, MET259, SER260, and SER277. These findings are consistent with previous studies that identified the same residues critical for the binding of T3 to TRα [59,60,61], with Chen, Z. F., et al. [62] and Song, Z., et al. [51] also reporting the involvement of SER277 in this binding. Similarly, OMC binding to zebrafish trα involved shared residues with T3, particularly PHE218 and MET259, mirroring observations in the human receptor TRα.

Regarding results for human TRβ, all compounds showed binding to the active centre of the receptors through key residues ILE 275, ILE276, LEU346, PHE269, PHE272, and THR273. OMC and T3 also shared additional binding residues ALA279, ASN331, HIS435, LEU330, MET310, MET313, and PHE455. These findings are consistent with previous studies, which have demonstrated the involvement of residues HIS435 [51,63,64,65], ASN331 [51,62], ALA279 [64,65], MET313 [64], ILE276, ALA279, and LEU330 [65] in the binding of T3 to TRβ. In zebrafish trβ, the binding of compounds was shared by almost all amino acid residues: ILE210, LEU264, LEU275, LEU280, MET376, PHE203, PHE206, PHE389, and THR207. These results align with those obtained by Jiao, F., et al. [66].

**Figure 7:**
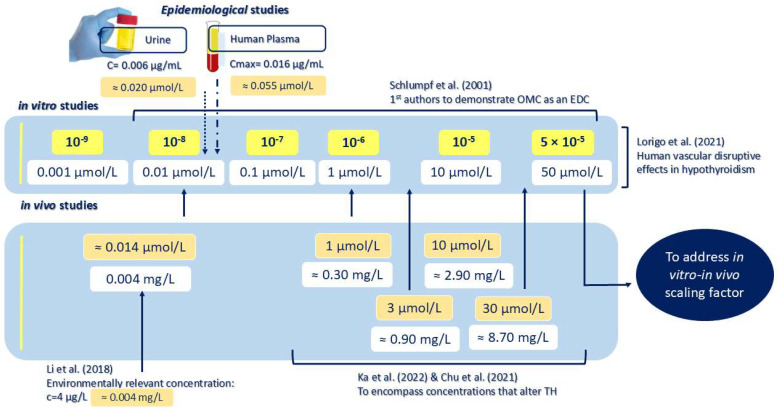
Illustrative representation of the most important literature-reported concentrations [21,22,23,71,85] of octyl methoxycinnamate (OMC) associated with thyroid disruption.

Therefore, our results indicate that, under steady-state conditions, OMC competes with T3 for binding to the active centre of TH nuclear receptors. This interaction may be explained by the structural similarity between TDCs and THs [67,68], particularly the shared double-ring chemical structure found in both OMC and T3. Previous studies have reported a down-regulation of the *trαa* and *trβ* genes after exposure to OMC, along with reduced larval survival rate in trαa−/− zebrafish exposed to ≥3 μmol/L of OMC [23]. Considering the good binding energies of OMC to TR obtained and the low k_i_ (in the order of 1–8 μmol/L) observed in this study, OMC appears to bind strongly to TRs, suggesting a high potential for inhibitory activity. Altogether, our data support the hypothesis that OMC can induce toxicity in both zebrafish and humans through TR-mediated disruptive effects. This “competitive” interaction between OMC and T3 will possibly prevent T3 from binding to TR/tr. Therefore, we assume that TR could combine with the TH response elements as monomers or homodimers and recruit corepressors. In these cases, gene transcription is repressed by the recruitment of deacetylated histones that promote a more compact and transcriptionally inactive chromatin structure [57,69]. This repression ultimately leads to reduced gene expression. Thus, OMC appears to be capable of reducing TH and to contribute to a hypothyroid-like state. This mechanism may explain the findings of Ka et al. (2022), who found a down-regulation of the *trαa* and *trβ* genes after exposure to OMC (10 and 30 µmol/L) [23]. In humans, TRβ1 is primarily responsible for mediating the negative feedback mechanism within the HPT axis, while TRα1 does not play any role in this regulatory loop [70]. Therefore, the decrease in *trβ* expression in zebrafish may trigger compensatory feedback mechanisms, prompting increased secretion of *crh* and *tsh* by the hypothalamus and pituitary gland, respectively, in an attempt to restore TH levels [23]. As T3 levels decrease in target cells, the body responds by increasing TSH secretion to stimulate additional T4 production and restore T3 levels, thereby completing the feedback loop. This hypothesis is supported by the literature, where exposure to OMC induces the up-regulation of *crh* [22], *tshβ* [23], *trh* [23], and *tshr* [22,23] in zebrafish larvae. In humans, our previous research demonstrated that OMC exposure disrupted vascular homeostasis in pregnant women with hypothyroidism, potentially involving TSHR and TRα1, as indicated by docking studies. The altered contractility patterns were observed after exposure to environmentally relevant concentrations of OMC (4 μg/L, corresponding to ~0.014 μmol/L [71]) and across a range of concentrations used for in vitro/in vivo extrapolation—scaling factor (0.001 to 50 μmol/L OMC) (Figure 7). Altogether, these results support our in silico findings suggesting OMC binding to crhr2, TSHR/tshr, and thyroid nuclear receptors (α and β) as the key mechanism to induce thyroid disruption. In this sense, OMC-induced endocrine disruption on the HPT axis is expected to induce harmful effects in zebrafish embryos and human development. In zebrafish developing embryos, thyroid hormone disruption has been associated with a range of outcomes, including developmental malformations [40], altered gene expression [36,72,73,74], hormonal imbalances [75], impaired eye development [72,73,75,76], swim bladder dysfunction [77], and behavioural alterations [40,72]. These findings reflect well-established TH-mediated processes and highlight the vulnerability of early development to TDC exposure (e.g., see reviews [78,79]). In humans, THs play an important role in maintaining vascular homeostasis [80,81], and their dysregulation can trigger several cardiovascular diseases. Multiple TDCs have been shown to have detrimental effects on human vasculature [21,82,83,84]. Regarding OMC, its thyroid-disrupting effects have already been reported in zebrafish (1 to 30 µmol/L of OMC [22,23]) and in the human vasculature of women with thyroid disorders (0.001 to 50 µmol/L of OMC [21]), which is in line with what was obtained in this investigation.

In summary, the primary mechanism by which OMC appears to affect the HPT axis is through direct binding to crhr2, TSHR/tshr, and TR/tr. In most simulations, OMC shared key amino acid interactions with T3 across different proteins involved in the HPT axis. Thus, and according to the literature, the OMC seems to induce primary hypothyroidism mimicking the endogenous hormone T3. The endocrine disruption of OMC on the HPT axis is evident by the action in TR/tr to reduce TH levels, leading two feedback negatives in the hypothalamus and pituitary (which leads to the increase in crhr2 in zebrafish and TSHR/tshr). In contrast, the OMC binding profile showed only limited similarity to that of PTU across the HPT axis proteins, suggesting that the MoA of OMC is not fully shared with this anti-thyroid drug. However, the direct binding of OMC to TPO and deiodinases, key proteins involved in the MoA of PTU [72,86,87], was not evaluated in this study. Therefore, the possibility of such binding interactions should not be excluded. Furthermore, existing in vitro/in vivo evidence in the literature suggests that OMC may also act on these proteins [19,20]. Therefore, future studies should clarify whether OMC also contributes to the reduction in TH levels.

This study represents the first in silico approach to comprehensively evaluate an entire endocrine axis, the HPT axis. The potential binding interactions of the UV-B filter and emerging contaminant OMC were explored across different proteins of the axis, from early hypothalamic–pituitary regulation (TRH/CRH/TSH) to specific nuclear receptor (TR) targets, including hormone transport (TTR). To date, published in silico studies have primarily focused on demonstrating the interaction of different EDCs with one, or at most two, components of the axis (e.g., TR: [59,62,64]; TTR: [88,89]). No previous research has addressed an endocrine axis with such depth and completeness. Importantly, this is also the first research evaluating the effects of TDC on the hypothalamus/pituitary gland, central regulators of the HPA, HPG, and HPT axes, thus providing valuable insights into the feedback mechanisms that may underlie endocrine disruption. Understanding the integration of these endocrine axes is fundamental, given the well-documented crosstalk demonstrated between them [69,90]. This is particularly important in an interspecies context, since zebrafish possess two distinct endocrine pathways influencing TH regulation (via trh and crh). Furthermore, this study examines the effects of a TDC—OMC—and compares it with two well-established reference substances—T3 and PTU—both of which are standardised models in the study of thyroid disruption [37,38]. The characterisation of the molecular targets and mechanisms of action of these compounds constitutes a significant advance in understanding how emerging contaminants like OMC affect thyroid function. This is especially relevant given the scarce and often inconclusive literature regarding OMC endocrine-disrupting potential. Currently, as thyroid disruption continues to raise concern in chemical risk assessment due to its profound implications for both human and environmental health, there is a pressing need for validated in silico and in vitro methods to identify and evaluate potential TDCs. For example, in environmental risk assessment, current standardised testing is largely limited to amphibian-based assays [38]. This research was designed to provide new insights into how molecular docking can serve as a new approach methodology (NAM) to evaluate TDCs within the integrated approaches to testing and assessment (IATA) framework. It aims to address critical gaps in the current testing landscape for thyroid disruption relevant to human and environmental health [91]. The MIEs, which are the starting points in AOPs, can be effectively evaluated by in silico studies, such as the one carried out in this research. Gaining knowledge at this early mechanistic level constitutes an added value for the understanding of the KEs of TDCs and consequent AOs, which are typically evaluated in subsequent ex vivo, in vitro, and in vivo studies [92]. This research aimed to fill a critical knowledge gap by providing a solid foundation on the MIEs and KEs at the beginning of an AOP, which help to explain and support the disruptive effects induced by OMC previously demonstrated in different in vitro/in vivo/ex vivo studies, in both humans [21] and zebrafish [22,23,24]. Notably, this study is the first to report the ADMET properties and in silico bioavailability triggers of a TDC, crucial for the construction of robust AOPs and understanding mechanisms of endocrine disruption [93]. Another important strength of this research is the interspecies comparison between humans and zebrafish, two models usually evaluated separately. Indeed, the zebrafish genome is fully sequenced and has about 70% gene homology with humans, including 84% of genes related to human diseases [9], along with a high degree of conservation in functional protein domains [94]. These features make zebrafish a powerful model for inferring potential human health effects. Developing zebrafish embryos are integrated into the internationally recognised OECD Test Guideline 236 for assessing contaminant toxicity and are also widely used as an in vivo model for assessing cardiotoxicity and the development of thyroid-related diseases [78]. In summary, our study addresses, for the first time and in an integrative way, the MIEs involved in thyroid endocrine disruption caused by OMC from a differentiating and innovative perspective, capable of clarifying how this TDC may act in humans and in the reference model, zebrafish.

## 4. Materials and Methods

### 4.1. Prediction of Pharmacokinetic Properties by Computational Analysis

**Figure 8:**
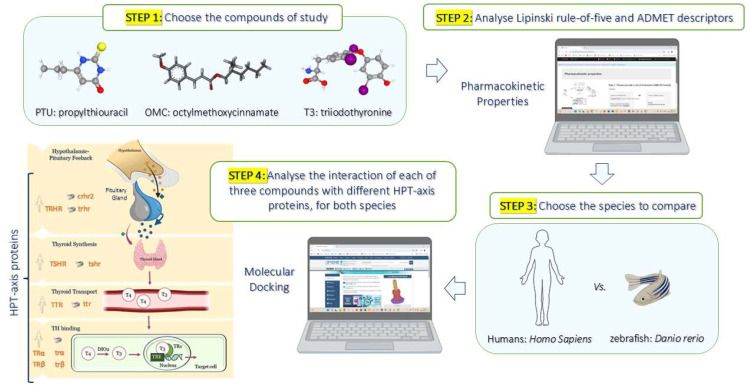
Illustrative representation of the methodology applied in this study. Firstly, the study compounds were defined as propylthiouracil (PTU), octyl methoxycinnamate (OMC), and triiodothyronine (T3), following a computational analysis of pharmacokinetic properties, including the Lipinski rule-of-five and ADMET (absorption, distribution, metabolism, excretion, and toxicity) descriptors. In the next step, the species to compare, *H. Sapiens* and *D. rerio*, were selected for molecular docking. These in silico simulations were designed to address different levels of the hypothalamus—pituitary—thyroid (HPT) axis, namely hypothalamic–pituitary feedback, synthesis, transport, and binding of thyroid hormones (THs) in both species. In this sense, the proteins chosen were thyroid-releasing hormone receptor (TRHR/trhr), corticotropin-releasing hormone receptor 2 (CRHR2/crhr2), transthyretin (TTR/ttr), and nuclear thyroid receptors alpha and beta (TRα/trα, TRβ/trβ). Finally, the interaction between each of the three compounds chosen and these different proteins of the HPT axis was performed for both species.

Chemical structures of triiodothyronine (T3, endogenous thyroid hormone), propylthiouracil (PTU, anti-thyroid drug), and octyl methoxycinnamate (OMC, environmental contaminant) were drawn in ACD/ChemSketch v.12.01, and all compounds were converted into canonical SMILES (Table S1). Pharmacokinetic properties of T3, PTU, and OMC were analysed by assessing Lipinski’s rule-of-five and ADMET (absorption, distribution, metabolism, excretion, and toxicity) descriptors using the algorithm protocol of pkCSM (http://biosig.unimelb.edu.au/pkcsm/prediction, accessed on 26 September 2025) (see the methodology details in Text S1 and Figure 8). Predicted molecule and ADMET properties of T3, PTU, and OMC are presented in Tables S2 and S3 and Texts S2 and S3. The results are discussed in Text S4.

### 4.2. In Silico Simulations by Molecular Docking

In silico simulations by molecular docking were performed (Autodock Tools v1.5.6 software) to assess the interaction of T3, PTU, and OMC between several HPT-axis-related proteins (see Text S5 and Table S4 in Supporting Information, and Figure 8). To analyse altered TH signalling, the proteins were chosen to include different cascade events of the HPT axis, such as thyroid stimulation (thyrotropin- and corticotropin-releasing hormone receptors and thyrotropin receptor), thyroid nuclear receptors (TRα and TRβ), and TH transport (transthyretin). OMC was drawn from the Database of Endocrine Disrupting Chemicals and Their Toxicity (DEDuCT) profiles (https://cb.imsc.res.in/deduct/, accessed on 5 February 2025)). The PubChem identities of the compounds and the CAS Registry Numbers of all ligands are presented in Table S5

Before the simulations, all ligands were subjected to Dock Prep using UCSF Chimera 1.15 software. In this process, the solvent and non-complex ions were removed. If alternate locations were available, only the highest-occupancy locations were kept. Selenomethionine (MSE) was changed to methionine (MET), bromo-UMP (5BU) was altered to UMP (U), methylselenyl-dUMP (UMS) was changed to UMP (U), and methylselenyl-dCMP (CSL) was changed to CMP (C). Moreover, hydrogens and charges were added.

## 5. Conclusions

**Figure 9:**
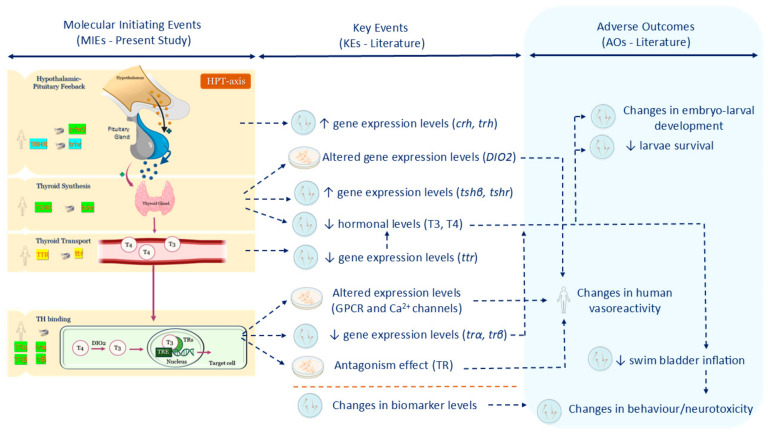
Illustrative diagram of the molecular initiating events (MIEs) of octyl methoxycinnamate (OMC) involved in thyroid disruption, as demonstrated in this study (**left side**). Green colour indicates the most favourable OMC bindings. Blue colour represents possible bindings, while yellow colour denotes bindings that are less physiologically probable. Down (↓) and up arrows (↑) represent decreases and increases, respectively. All MIEs analysed were related to the key events (KEs) and adverse outcomes (AOs) reported in the literature for exposure to OMC (**on the centre and right sides**). Based on previous data [19,20,21,22,23,24,95,96,97,98,99,100,101].

In conclusion, this investigation suggests that OMC acts at different levels of the HPT axis, with target-specific and specie-dependent mechanisms. OMC appears to interfere with hormonal synthesis via crhr2 and TSHR/tshr (hypothalamic–pituitary feedback) and impairs TH action at target cells through interaction with TRs/trs. Overall, these actions of OMC appear to lead to hypothyroidism. Physiologically, the literature indicates that the human HPT axis tries to compensate the TH decrease, increasing the expression of TRH and TSH to recover levels. These events are in accordance with the obtained favourable binding of OMC to zebrafish crhr2 (which is the main regulator of the HPT axis in this specie) and TSHR/tshr (human/zebrafish). However, the favourable binding of OMC to TR/tr (human/zebrafish) reveals an anti-thyroid action, promoting TH reduction, as reported in previous in vitro/in vivo studies [22,23] (Figure 9). Therefore, OMC at the different levels of HPT axis promotes typical negative feedback similar to the same primary hypothyroidism condition. This investigation provides new in silico insights into the thyroid-disrupting potential of OMC across species, reinforcing its classification as a TDC. Additional experimental studies must be conducted to confirm the mechanisms and relevance of OMC-induced thyroid disruption.

## Figures and Tables

**Table 1:** Lowest Gibbs free energy (estimated as ΔG in kcal/mol), inhibition constant (estimated as k_i_ in µmol/L), and hydrogen (H) bridges were calculated from molecular docking studies for each ligand. The bold emphasises the most favourable ligand binding interaction with each target protein. N/A—not applicable.

Protein	Compound	ΔG (kcal/mol)	k_i_ (µmol/L)	H-Bridges	Organism
TRHR	T3	**−8.01**	1.34	ALA78 (2.454 Å)	*H. sapiens* (Humans)
	PTU	−4.06	1060.00	N/A	
	OMC	−5.21	150.97	N/A	
trhr	T3	**−6.86**	9.33	N/A	*D. rerio* (Zebrafish)
	PTU	−4.53	477.19	N/A	
	OMC	−5.77	59.27	N/A	
crhr2	T3	−0.13	809,260.00	N/A	*D. rerio* (Zebrafish)
	PTU	−5.50	92.27	N/A	
	OMC	**−6.45**	18.6	N/A	
TSHR	T3	−4.50	504.63	GLN489 (2.631 Å)	*H. sapiens* (Humans)
	PTU	−4.66	385.30	N/A	
	OMC	**−5.68**	68.23	N/A	
tshr	T3	−5.60	78.04	N/A	*D. rerio* (Zebrafish)
	PTU	−4.41	585.23	N/A	
	OMC	**−5.86**	50.89	N/A	
TTR	T3	**−5.75**	60.88	N/A	*H. sapiens* (Humans)
	T4	−5.06	196.03	N/A	
	PTU	−3.44	3010.00	N/A	
	OMC	−3.66	2090.00	N/A	
ttr	T3	**−5.88**	49.31	(LEU132, 2.426 Å and SER137, 2.076 Å)	*D. rerio* (Zebrafish)
	T4	−5.11	179.92	N/A	
	PTU	−3.60	2300.00	(ALA130, 2.270 Å)	
	OMC	−3.53	2600.00	N/A	
TRα	T3	−4.54	468.17	ILE378 (2.203 Å)	*H. sapiens* (Humans)
	PTU	−4.63	407.09	N/A	
	OMC	**−6.92**	8.49	N/A	
trα	T3	−6.42	19.81	N/A	*D. rerio* (Zebrafish)
	PTU	−5.23	146.05	N/A	
	OMC	**−7.83**	1.83	N/A	
TRβ	T3	−6.48	17.77	N/A	*H. sapiens* (Humans)
	PTU	−5.39	112.22	(THR329, 2.323 Å)	
	OMC	**−7.88**	1.68	N/A	
trβ	T3	−5.95	43.82	(MET247, 1.872 Å)	*D. rerio* (Zebrafish)
	PTU	−5.50	92.64	(ASN265, 2.027 Å)	
	OMC	**−8.13**	1.09	N/A	

## Data Availability

The original contributions presented in this study are included in the article/Supplementary Materials. Further inquiries can be directed to the corresponding authors.

## Supplementary Materials

The following supporting information can be downloaded at: https://www.mdpi.com/article/10.3390/ph18121897/s1, Figure S1: Chemical Structures of triiodothyronine (T3), octyl methoxycinnamate (OMC), and propylthiouracil (PTU) drawn in ACD/ChemSketch v.12.01; Table S1: Canonical SMILES of compounds used in this study; Text S1: Applied methodology using the algorithm protocol of pkCSM to assess the Lipinski rule-of-five and ADMET (absorption, distribution, metabolism, excretion, and toxicity) descriptors [93,102]; Table S2: Predicted molecule properties of compounds used in this study; Table S3: Predicted ADMET properties of compounds used in this study; Text S2: Results of Lipinski’s rule-of-five parameters; Text S3: Results of ADMET descriptors; Text S4: Discussion of ADMET and Lipinski’s rule-of-five data [17,18,22,23,30,31,33,35,76,103,104,105,106,107,108,109,110,111,112,113]; Text S5: Applied methodology to docking simulations [114,115,116,117,118,119,120,121,122,123]; Table S4: UniProt/AlphaFold (AF) accession, protein and gene names, % of identity between the species analysed and organism test; Table S5: Nomenclature, ligand and PubChem IDs, and Chemical Abstracts Service Registry Number (CASRN) of ligands; Figure S2: 3D Ramachandran plot from thyrotropin-releasing hormone receptor (TRHR, human) of six distinct categories: (a) general case (Ala and remaining 15 amino acids), (b) Gly, (c) Val/Ile, (d) pre-Pro, (e) trans-Pro, and (f) cis-Pro. Bars represent the frequency of torsion angles; Figure S3: 3D Ramachandran plot from thyrotropin-releasing hormone receptor (trhrb, zebrafish) of six distinct categories: (a) general case (Ala and remaining 15 amino acids), (b) Gly, (c) Val/Ile, (d) pre-Pro, (e) trans-Pro, and (f) cis-Pro. Bars represent the frequency of torsion angles; Figure S4: 3D Ramachandran plot from corticotropin-releasing hormone receptor 2 (crhr2, zebrafish) of six distinct categories: (a) general case (Ala and remaining 15 amino acids), (b) Gly, (c) Val/Ile, (d) pre-Pro, (e) trans-Pro, and (f) cis-Pro. Bars represent the frequency of torsion angles; Figure S5: 3D Ramachandran plot from thyrotropin# receptor (TSHR, humans) of six distinct categories: (a) general case (Ala and remaining 15 amino acids), (b) Gly, (c) Val/Ile, (d) pre-Pro, (e) trans-Pro, and (f) cis-Pro. Bars represent the frequency of torsion angles; Figure S6: 3D Ramachandran plot from thyrotropin# receptor (tshr, zebrafish) of six distinct categories: (a) general case (Ala and remaining 15 amino acids), (b) Gly, (c) Val/Ile, (d) pre-Pro, (e) trans-Pro, and (f) cis-Pro. Bars represent the frequency of torsion angles; Figure S7: 3D Ramachandran plot from transthyretin (TTR, humans) of six distinct categories: (a) general case (Ala and remaining 15 amino acids), (b) Gly, (c) Val/Ile, (d) pre-Pro, (e) trans-Pro, and (f) cis-Pro. Bars represent the frequency of torsion angles; Figure S8: 3D Ramachandran plot from transthyretin (ttr, zebrafish) of six distinct categories: (a) general case (Ala and remaining 15 amino acids), (b) Gly, (c) Val/Ile, (d) pre-Pro, (e) trans-Pro, and (f) cis-Pro. Bars represent the frequency of torsion angles; Figure S9: 3D Ramachandran plot from thyroid hormone receptor alpha (THRA, humans) of six distinct categories: (a) general case (Ala and remaining 15 amino acids), (b) Gly, (c) Val/Ile, (d) pre-Pro, (e) trans-Pro, and (f) cis-Pro. Bars represent the frequency of torsion angles; Figure S10: 3D Ramachandran plot from thyroid hormone receptor alpha-A (thraa, zebrafish) of six distinct categories: (a) general case (Ala and remaining 15 amino acids), (b) Gly, (c) Val/Ile, (d) pre-Pro, (e) trans-Pro, and (f) cis-Pro. Bars represent the frequency of torsion angles; Figure S11: 3D Ramachandran plot from thyroid hormone receptor beta (THRB, humans) of six distinct categories: (a) general case (Ala and remaining 15 amino acids), (b) Gly, (c) Val/Ile, (d) pre-Pro, (e) trans-Pro, and (f) cis-Pro. Bars represent the frequency of torsion angles; Figure S12: 3D Ramachandran plot from thyroid hormone receptor beta (thrb, zebrafish) of six distinct categories: (a) general case (Ala and remaining 15 amino acids), (b) Gly, (c) Val/Ile, (d) pre-Pro, (e) trans-Pro, and (f) cis-Pro. Bars represent the frequency of torsion angles; Figure S13: 3D-representation of preferred conformation and interactions with amino acid residues of the complex between the ligands propylthiouracil (PTU, blue), octyl methoxycinnamate (OMC, orange), and triiodothyronine (T3, grey) with thyrotropin-releasing hormone receptor from (A) humans and (B) zebrafish, using Autodock; Figure S14: Atomic interactions by Discovery Studio of the complex between the ligands propylthiouracil (PTU, blue), octyl methoxycinnamate (OMC, orange), and triiodothyronine (T3, grey) with thyrotropin-releasing hormone receptor from (A) humans and (B) zebrafish; Figure S15: Atomic interactions by Discovery Studio of the complex between the ligands propylthiouracil (PTU, blue), octyl methoxycinnamate (OMC, orange), and triiodothyronine (T3, grey) with corticotropin-releasing hormone receptor from (B) zebrafish; Figure S16: 3D-representation of preferred conformation and interactions with amino acid residues of the complex between the ligands propylthiouracil (PTU, blue), octyl methoxycinnamate (OMC, orange), and triiodothyronine (T3, grey) with thyroid-stimulating hormone receptor (or thyrotropin receptor) from (B) zebrafish, using Autodock; Figure S17: Atomic interactions by Discovery Studio of the complex between the ligands propylthiouracil (PTU, blue), octyl methoxycinnamate (OMC, orange), and triiodothyronine (T3, grey) with thyroid-stimulating hormone receptor (or thyrotropin receptor) from (B) zebrafish; Figure S18: 3D-representation of preferred conformation and interactions with amino acid residues of the complex between the ligands propylthiouracil (PTU, blue), octyl methoxycinnamate (OMC, orange), and triiodothyronine (T3, grey) with transthyretin from (A) humans and (B) zebrafish, using Autodock; Figure S19: Atomic interactions by Discovery Studio of the complex between the ligands propylthiouracil (PTU, blue), octyl methoxycinnamate (OMC, orange), triiodothyronine (T3, grey), and tetraiodothyronine (T4, yellow) with transthyretin from (A) humans and (B) zebrafish; Figure S20: 3D-representation of preferred conformation and interactions with amino acid residues of the complex between the ligands propylthiouracil (PTU, blue), octyl methoxycinnamate (OMC, orange), and triiodothyronine (T3, grey) with thyroid hormone receptor alpha from (A) humans and (B) zebrafish, using Autodock; Figure S21: Atomic interactions by Discovery Studio of the complex between the ligands propylthiouracil (PTU, blue), octyl methoxycinnamate (OMC, orange), and triiodothyronine (T3, grey) with thyroid hormone receptor alpha from (A) humans and (B) zebrafish; Figure S22: 3D-representation of preferred conformation and interactions with amino acid residues of the complex between the ligands propylthiouracil (PTU, blue), octyl methoxycinnamate (OMC, orange), and triiodothyronine (T3, grey) with thyroid hormone receptor beta from (A) humans and (B) zebrafish, using Autodock; Figure S23: Atomic interactions by Discovery Studio of the complex between the ligands propylthiouracil (PTU, blue), octyl methoxycinnamate (OMC, orange), and triiodothyronine (T3, grey) with thyroid hormone receptor beta from (A) humans and (B) zebrafish.
