# Supplementary material for: In Silico Identification of Molecular Interactions of the Emerging Contaminant Octyl Methoxycinnamate (OMC) on HPT Axis: Implications for Humans and Zebrafish
Source: Pharmaceuticals (Basel). 2025 Dec 16;18(12):1897. doi: 10.3390/ph18121897 (PMC12736302; doi:10.3390/ph18121897)
Supplement: Supplementary file 1 [file pharmaceuticals-18-01897-s001.zip › pharmaceuticals-4017431-supplementary.pdf]

Supplementary Data

In silico identification of molecular interactions of the emerging contaminant octylmethoxycinnamate (OMC) on HPT-axis: implications for humans and zebrafish

Margarida Lorigo [a,b,c] *(margarida.lorigo@gmail.com), Luiza Breitenfeld* [a,b] *(luiza@fcsaude.ubi.pt), Marta S. Monteiro* [c] *(mmonteiro@ua.pt), Amadeu M.V.M. Soares* [c] *(asoares@ua.pt), Carla Quintaneiro* [c] *(cquintaneiro@ua.pt), and Elisa Cairrao* [a,b,*] *(ecairrao@fcsaude.ubi.pt)*

[a] RISE-Health, Department of Medical Sciences, Faculty of Health Sciences, University of Beira Interior, 6200-506, Covilhã, Portugal.

[b] FCS-UBI, Faculty of Health Sciences, University of Beira Interior, 6200-506, Covilhã, Portugal.

[c] Department of Biology & CESAM (Centre for Environmental and Marine Studies), University of Aveiro, 3810-193, Aveiro, Portugal.

[*] **Corresponding author:**

Elisa Cairrao, PhD

RISE-Health, Department of Medical Sciences, Faculty of Health Sciences, University of Beira Interior, 6200-506, Covilhã, Portugal

Tel.: +351-275-329049 | Fax: +351-275-329099

e-mail: ecairrao@fcsaude.ubi.pt

ORCID iD: https://orcid.org/0000-0002-4823-5701

Number of pages: 41

Number of Text Sections: 5

Number of Figures: 23

Number of Tables: 5

CONTENTS

Figure S1. Chemical Structures of triiodothyronine (T3), octylmethoxycinnamate (OMC), and propylthiouracil (PTU) drawn in ACD/ChemSketch v.12.01.

Table S1. Canonical SMILES of compounds used in this study.

Text S1. Applied methodology using the algorithm protocol of pkCSM to assess the Lipinski rule-of-five and ADMET (Absorption, distribution, metabolism, excretion, and toxicity) descriptors.

Table S2. Predicted Molecule Properties of compounds used in this study.

Table S3. Predicted ADMET properties of compounds used in this study.

Text S2. Results of Lipinski's rule-of-five parameters

Text S3. Results of ADMET descriptors.

Text S4. Discussion of ADMET and Lipinski-rule of five data

Text S5. Applied methodology to docking simulations.

Table S4. UniProt / AlphaFold (AF) Accession, Protein and Gene names, % of identity between the species analysed and organism test.

Table S5. Nomenclature, ligand and PubChem IDs, and Chemical Abstracts Service Registry Number (CASRN) of ligands.

Figure S2. 3D Ramachandran plot from Thyrotropin-releasing hormone receptor (TRHR, human) of six distinct categories: **(a)** general case (Ala and remaining 15 amino acids), **(b)** Gly, **(c)** Val/Ile, **(d)** pre-Pro, **(e)** trans-Pro & **(f)** cis-Pro. Bars represent the frequency of torsion angles.

Figure S3. 3D Ramachandran plot from Thyrotropin-releasing hormone receptor (trhrb, zebrafish) of six distinct categories: **(a)** general case (Ala and remaining 15 amino acids), **(b)** Gly, **(c)** Val/Ile, **(d)** pre-Pro, **(e)** trans-Pro & **(f)** cis-Pro. Bars represent the frequency of torsion angles.

Figure S4. 3D Ramachandran plot from Corticotropin releasing hormone receptor 2 (crhr2, zebrafish) of six distinct categories: **(a)** general case (Ala and remaining 15 amino acids), **(b)** Gly, **(c)** Val/Ile, **(d)** pre-Pro, **(e)** trans-Pro & **(f)** cis-Pro. Bars represent the frequency of torsion angles.

Figure S5. 3D Ramachandran plot from Thyrotropin# receptor (TSHR, humans) of six distinct categories: (a) general case (Ala and remaining 15 amino acids), (b) Gly, (c) Val/Ile, (d) pre-Pro, (e) trans-Pro & (f) cis-Pro. Bars represent the frequency of torsion angles.

Figure S6. 3D Ramachandran plot from Thyrotropin# receptor (tshr, zebrafish) of six distinct categories: (a) general case (Ala and remaining 15 amino acids), (b) Gly, (c) Val/Ile, (d) pre-Pro, (e) trans-Pro & (f) cis-Pro. Bars represent the frequency of torsion angles.

Figure S7. 3D Ramachandran plot from Transthyretin (TTR, humans) of six distinct categories: (a) general case (Ala and remaining 15 amino acids), (b) Gly, (c) Val/Ile, (d) pre-Pro, (e) trans-Pro & (f) cis-Pro. Bars represent the frequency of torsion angles.

Figure S8. 3D Ramachandran plot from Transthyretin (ttr, zebrafish) of six distinct categories: (a) general case (Ala and remaining 15 amino acids), (b) Gly, (c) Val/Ile, (d) pre-Pro, (e) trans-Pro & (f) cis-Pro. Bars represent the frequency of torsion angles.

Figure S9. 3D Ramachandran plot from Thyroid hormone receptor alpha (THRA, humans) of six distinct categories: (a) general case (Ala and remaining 15 amino acids), (b) Gly, (c) Val/Ile, (d) pre-Pro, (e) trans-Pro & (f) cis-Pro. Bars represent the frequency of torsion angles.

Figure S10. 3D Ramachandran plot from Thyroid hormone receptor alpha-A (thraa, zebrafish) of six distinct categories: (a) general case (Ala and remaining 15 amino acids), (b) Gly, (c) Val/Ile, (d) pre-Pro, (e) trans-Pro & (f) cis-Pro. Bars represent the frequency of torsion angles.

Figure S11. 3D Ramachandran plot from Thyroid hormone receptor beta (THRB, humans) of six distinct categories: (a) general case (Ala and remaining 15 amino acids), (b) Gly, (c) Val/Ile, (d) pre-Pro, (e) trans-Pro & (f) cis-Pro. Bars represent the frequency of torsion angles.

Figure S12. 3D Ramachandran plot from Thyroid hormone receptor beta (thrb, zebrafish) of six distinct categories: (a) general case (Ala and remaining 15 amino acids), (b) Gly, (c) Val/Ile, (d) pre-Pro, (e) trans-Pro & (f) cis-Pro. Bars represent the frequency of torsion angles.

Figure S13. 3D-representation of preferred conformation and interactions with amino acid residues of the complex between the ligands propylthiouracil (PTU, blue), octylmethoxycinnamate (OMC, orange) and triiodothyronine (T3, grey) with Thyrotropin-Releasing Hormone Receptor from (A) humans and (B) zebrafish, using Autodock.

Figure S14. Atomic interactions type by Discovery Studio of the complex between the ligands propylthiouracil (PTU, blue), octylmethoxycinnamate (OMC, orange) and triiodothyronine (T3, grey) with Thyrotropin-Releasing Hormone Receptor from **(A)** humans and **(B)** zebrafish.

Figure S15. Atomic interactions type by Discovery Studio of the complex between the ligands propylthiouracil (PTU, blue), octylmethoxycinnamate (OMC, orange) and triiodothyronine (T3, grey) with Corticotropin-Releasing Hormone Receptor from **(B)** zebrafish.

Figure S16. 3D-representation of preferred conformation and interactions with amino acid residues of the complex between the ligands propylthiouracil (PTU, blue), octylmethoxycinnamate (OMC, orange) and triiodothyronine (T3, grey) with Thyroid Stimulating Hormone Receptor (or Thyrotropin receptor) from **(B)** zebrafish, using Autodock.

Figure S17. Atomic interactions type by Discovery Studio of the complex between the ligands propylthiouracil (PTU, blue), octylmethoxycinnamate (OMC, orange) and triiodothyronine (T3, grey) with Thyroid Stimulating Hormone Receptor (or Thyrotropin receptor) from **(B)** zebrafish.

Figure S18. 3D-representation of preferred conformation and interactions with amino acid residues of the complex between the ligands propylthiouracil (PTU, blue), octylmethoxycinnamate (OMC, orange) and triiodothyronine (T3, grey) with Transthyretin from **(A)** humans and **(B)** zebrafish, using Autodock.

Figure S19. Atomic interactions type by Discovery Studio of the complex between the ligands propylthiouracil (PTU, blue), octylmethoxycinnamate (OMC, orange), triiodothyronine (T3, grey) and tetraiodothyronine (T4, yellow) with Transthyretin from **(A)** humans and **(B)** zebrafish.

Figure S20. 3D-representation of preferred conformation and interactions with amino acid residues of the complex between the ligands propylthiouracil (PTU, blue), octylmethoxycinnamate (OMC, orange) and

triiodothyronine (T3, grey) with Thyroid Hormone Receptor alpha from **(A)** humans and **(B)** zebrafish, using Autodock.

Figure S21. Atomic interactions type by Discovery Studio of the complex between the ligands propylthiouracil (PTU, blue), octylmethoxycinnamate (OMC, orange) and triiodothyronine (T3, grey) with Thyroid Hormone Receptor alpha from **(A)** humans and **(B)** zebrafish.

Figure S22. 3D-representation of preferred conformation and interactions with amino acid residues of the complex between the ligands propylthiouracil (PTU, blue), octylmethoxycinnamate (OMC, orange) and triiodothyronine (T3, grey) with Thyroid Hormone Receptor beta from **(A)** humans and **(B)** zebrafish, using Autodock.

Figure S23. Atomic interactions type by Discovery Studio of the complex between the ligands propylthiouracil (PTU, blue), octylmethoxycinnamate (OMC, orange) and triiodothyronine (T3, grey) with Thyroid Hormone Receptor beta from **(A)** humans and **(B)** zebrafish.

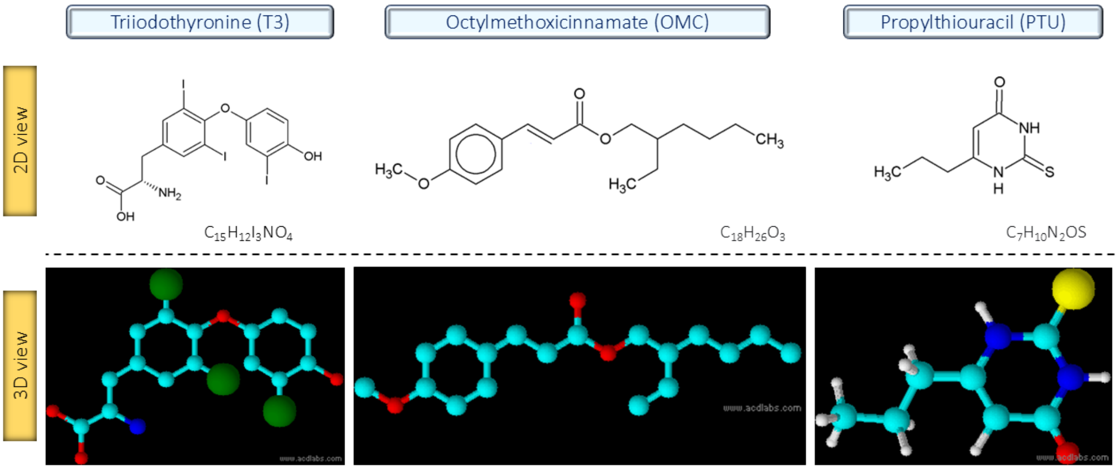

Figure S1. Chemical Structures of triiodothyronine (T3), octylmethoxycinnamate (OMC), and propylthiouracil (PTU) drawn in ACD/ChemSketch v.12.01. The superior panel shows a 2D view, and the inferior panel illustrates a 3D view.

Table S1. Canonical SMILES of compounds used in this study (T3: triiodothyronine; PTU: propylthiouracil; and OMC: octylmethoxycinnamate).

Compound	SMILES
Triiodothyronine	IC1=CC(C[C@H](N)C(O)=O)=CC(I)=C1OC2=CC(I)=C(O)C=C2
Propylthiouracil	O=C(N1)C=C(CCC)NC1=S
Octylmethoxycinnamate	COC1=CC=C(/C=C/C(OCC(CC)CCCC)=O)C=C1

Text S1. Applied methodology using the algorithm protocol of pkCSM to assess the Lipinski rule-of-five and ADMET (Absorption, distribution, metabolism, excretion, and toxicity) descriptors.

To predict the bioavailability of compounds in TH signalling, rule of five of Lipinski was used [1]. This assessment considered only the passive-diffusive component of molecular bioavailability concerning the deregulation of TH signalling (direct or indirect), ignoring active transport pathways for TH-related targets, if present [2]. According to the Lipinski rule of five, compounds with low bioavailability will have poor absorption/membrane permeation evaluating the following criteria: 1) hydrogen bond donors (HBD)>5; 2) hydrogen bond acceptors (HBA)>10; 3) molecular weight (MW)> 500 Da (1 Dalton=1g/mol), and 4) logarithmic octanol/water partition coefficient (log K_{ow})>5 and 5) number of rotatable bonds >5. Poor membrane absorption/permeation is predicted if at least two criteria are met. In the method, the "5" results from the fact that there are 5 limits and that the numerical values for them are 5 (#HBD, log K_{ow}, number of rotatable bonds) or multiples of 5 (#HBA, MW). Concerning the ADMET prediction, the parameters were calculated and checked for compliance with their standard ranges. The absorption of the drugs was predicted based on colon cancer cell line Caco-2 permeability (indicating its membrane permeability), water solubility, intestinal absorption (human), P-glycoprotein substrate or inhibitor, and skin permeability. The volume of distribution (VDss) (human), fraction unbound (human), blood-brain barrier (BBB), and central nervous system (CNS) permeability confers the distribution data of the drug. The metabolism depends on the CYP models for cytochrome P450 inhibitors or CYP2D6/CYP3A4 substrate. Excretion properties were predicted based on the renal Organic Cation Transporter 2 (OCT2) substrate and total clearance. Toxicity of drugs was predicted based on Rat LD50, AMES toxicity, *Tetrahymena pyriformis* toxicity, Minnow toxicity, Maximum Tolerated Dose, Oral Rat Chronic Toxicity, Hepatotoxicity, Skin sensitisation and hERG I and II Inhibitors.

Table S2. Predicted Molecule Properties of compounds used in this study (T3: triiodothyronine; PTU: propylthiouracil and OMC: octylmethoxycinnamate).

Properties	T3	PTU	OMC
Molecular Weight (g/mol)	650.976	170.237	290.403
LogP	3.9527	1.38499	4.468
#Rotatable Bonds	5	2	9
#Acceptors	4	2	3
#Donors	3	2	0
Surface Area	173.572	69.486	127.510

Table S3. Predicted ADMET properties of compounds used in this study (T3: triiodothyronine; PTU: propylthiouracil; and OMC: octylmethoxycinnamate). *To facilitate identification between the chemicals, colour coding was applied: pink for highly positive, yellow for faint positive, and green for negative.* *Legend:* ADMET – Absorption, distribution, metabolism, excretion, and toxicity; AMES – assay of the ability of a chemical compound to induce mutations in DNA; BBB – blood-brain barrier; CNS – central nervous system; Fu, fraction unbound; Kp – skin permeability constant; LD – lethal dose; LOAEL – lowest observed adverse effect level; Papp – apparent permeability coefficient; PS – permeability-surface area; *T. pyriformis* – *Tetrahymena pyriformis*.

Properties	T3	PTU	OMC
**Absorption**			
Water solubility (log mol/L)	-3.218	-1.447	-5.217
Caco2 permeability (log Papp in 10^{-6} cm/s)	0.803	1.213	1.625
Intestinal absorption (human, % Absorbed)	63.406	92.309	94.938
Skin Permeability (log Kp)	-2.735	-2.921	-2.438
P-glycoprotein substrate	Yes	No	No
P-glycoprotein I inhibitor	No	No	Yes
P-glycoprotein II inhibitor	No	No	No
**Distribution**			
VDss (human, log L/Kg)	-0.127	-0.113	0.269
Fraction unbound (human, Fu)	0.212	0.614	0.014
BBB permeability (log BB)	-1.486	-0.321	-0.106
CNS permeability (log PS)	-2.39	-2.871	-1.848
**Metabolism**			
CYP2D6 substrate	Yes	No	No
CYP3A4 substrate	No	No	Yes
CYP1A2 inhibitor	No	No	Yes
CYP2C19 inhibitor	No	No	Yes
CYP2C9 inhibitor	No	No	No
CYP2D6 inhibitor	No	No	No
CYP3A4 inhibitor	No	No	No
**Excretion**			
Total Clearance (log ml/min/Kg)	-0.146	0.072	0.92
Renal OCT2 substrate	No	No	No
**Toxicity**			
AMES toxicity	No	No	No
Max. tolerated dose (human, log mg/kg/day)	-1.053	0.781	0.95
hERG I inhibitor	No	No	No
hERG II inhibitor	No	No	No
Oral Rat Acute Toxicity (LD_{50}, mol/Kg)	2.76	2.61	1.687
Oral Rat Chronic Toxicity (LOAEL, log mg/kg_bw/day)	1.601	1.334	2.306
Hepatotoxicity	No	Yes	Yes
Skin Sensitisation	No	Yes	Yes
T. pyriformis toxicity (log µg/L)	0.285	-0.191	2.006
Minnow toxicity (log mM)	-0.259	2.272	-0.747

Text S2. Results of Lipinski's rule-of-five parameters

Regarding the parameters of Lipinski's rule-of-five, PTU does not met any criteria, while T3 and OMC met two or one of them, respectively (**Table S2**). The MW of T3 is higher than 500 g/mol, and the number of rotatable is equal to 5. In the case of OMC, the number of rotatable is higher than 5. Therefore, PTU is predicted to have good membrane absorption/permeation and high bioavailability. Contrarily, the T3 will have more difficulty permeating the membrane and low bioavailability, since two of the criteria are met. The OMC only meets one of the criteria, so it is predicted that it can permeate the membrane more easily than T3, but with greater difficulty than PTU.

Text S3. Results of ADMET descriptors.

Absorption: The predicted values for T3 (-3.218 mol/L), PTU (-1.447 mol/L), and OMC (-5.217 mol/L) indicate that PTU is the most soluble in water at 25ºC. These results agree with the physicochemical properties of the molecules under study since PTU is hydrosoluble while T3 and OMC are liposoluble, indicating that they are less well absorbed than PTU. The Caco-2 permeability values suggest that the oral absorption of PTU and OMC is expected to occur with high permeability since the values are higher than 0.90 cm/s; however, it seems to be higher for OMC (OMC=1.625 cm/s *vs.* PTU=1.213). The same was not valid for T3, whose value was less than 0.90 cm/s (T3=0.803 cm/s). In agreement, PTU and OMC are expected to be easily absorbed by the human intestine with a % of around 90%, and T3 with a lower % (approximately 60%). Regarding skin permeability, it seems that OMC has a relatively low skin permeability since logKp>-2.5 (OMC=-2.438 Kp). The same was not observed for T3 (-2.735 Kp) and PTU (-2.921 Kp). None of the drugs under study appear to be P-glycoprotein II inhibitors. However, OMC may be a P-glycoprotein I inhibitor and T3 a P-glycoprotein substrate.

Distribution: The volume of distribution is considered low when the value is less than 0.15 log VDss and high when log VDss >0.45. Thus, T3 and PTU have low VDss of -0.127 log L/Kg (T3) and -0.113 log L/Kg (PTU), while OMC (0.269 log L/Kg) has normal VDss. The predicted fraction that would be unbound in plasma for the three compounds would be 0.212 Fu (T3), 0.614 Fu (PTU), and 0.014 Fu (OMC), indicating that OMC will have the highest probability of binding to serum proteins and will therefore cross cell membranes less efficiently or diffuse with greater difficulty. PTU and OMC seem able to distribute themselves throughout the brain, but they have difficulty crossing the BBB since both have a -1<logBB<0.3 (PTU=-0.321 log BB and OMC=-0.106 log BB). Despite this, OMC seems to be able to penetrate the CNS since it presented a logPS>-2 (OMC=-1.848 logPS). The T3 seems poorly distributed to the brain (logBB <-1, logBB= -1.486).

Metabolism: The T3 may be a CYP2D6 substrate and can be metabolized by cytochrome P450. OMC may be a potential cytochrome P450 inhibitor for the CYP1A2 and CYP2C19 isoforms and is expected to be metabolized via the CYP3A4 substrate. No positive association was observed for PTU to inhibit cytochrome P450 or be metabolized by it.

Excretion: Total Clearance of OMC (0.92 log ml/min/Kg) was considerably higher than that of T3 (-0.146 log ml/min/Kg) and PTU (0.072 log ml/min/Kg). None of the drugs under study seems expected to be a renal OCT2 substrate.

Toxicity: None of the compounds under study is expected to be mutagenic. The maximum recommended tolerated dose for PTU=0.781 and OMC=0.95 log mg/kg/day was high as it was higher than 0.477 log mg/kg/day, indicating an estimate of the toxic dose limit of these drugs in humans. None of the drugs under analysis proved to be a probable hERG I/II inhibitor. The predicted LD50 for T3 and PTU were higher than for OMC, indicating that these compounds appear less toxic. Surprisingly, the LOAEL prediction indicated that PTU and T3 require a lower dose to observe an adverse effect compared to OMC. PTU and OMC are potential hepatotoxic molecules that appear to be associated with skin sensitization.

Predictions of *T. pyriformis* toxicity and *minnow toxicity* showed that OMC seems to be the most toxic of the three compounds analysed.

Text S4. Discussion of ADMET and Lipinski-rule of five data

The extensive application of UV filters in daily human life has led to continuous exposure to these contaminants through different sources and exposure pathways. Furthermore, the extensive production associated with the lack of capacity of wastewater treatment plants to eliminate these wastes increases the exposure of aquatic organisms to these compounds. Worryingly, the prevalence of OMC (~90%) and its capacity for bioaccumulation and biomagnification in the food chain highlight the need to evaluate the adverse effects of OMC for human and environmental protection. Early periods of development, including pregnancy, are more prone to the development of endocrine disruption [3]. Pregnant women apply PCPs containing UV filters daily. The main pathway of exposure to OMC is dermal contact [4], where its entry into the systemic circulation is facilitated (without initial hepatic metabolism) [5]. Our ADMET data suggest an absorption of OMC, which agrees with previous investigations [6, 7]. At the distribution level, our data suggests that the OMC can penetrate the CNS since it presented a logPS>-2 (OMC=-1.848 logPS). In the literature, the effects of OMC on the CNS have been reported *in vivo* [8-11]. Regarding metabolism, cytochrome P450 is a vital enzyme complex as a defence mechanism against EDCs, metabolizing them mainly through phase I reactions. Our ADMET data suggest that OMC may be a potential cytochrome P450 inhibitor for the CYP1A2 and CYP2C19 isoforms and is expected to be metabolized via the CYP3A4 substrate. Total clearance of OMC (0.92 log ml/min/Kg) was higher, agreeing with previous studies where OMC was detected in human urine [6]. Being rapidly absorbed by the skin, OMC reaches the systemic circulation in its unchanged form; therefore, it is expected to accumulate in various tissues. The bioaccumulation of OMC has not yet been reported in the human placenta [12], but this is not unreasonable since the detection of OMC has been demonstrated in breast milk samples [13, 14]. Furthermore, the role of obesogenic EDC has also been suggested for OMC [15], and maternofoetal transfer has been demonstrated in non-human mammalian animals [16]. In this sense, studying the potentially harmful effects of the OMC in this sensitive development window constitutes a challenging task, as very little is known about this topic. To facilitate this task, we recently reviewed the effects of OMC on human health [17] and on aquatic organisms [18]. In the present study, emphasis was placed on using developing zebrafish embryos as a model for evaluating mammalian thyroid disruption following exposure to OMC. This choice was due to the high similarity in physiology and development between the two models. The physiological responses of embryos/larvae (e.g., hatching, malformations, size, and heart rate) have often been used in the developmental toxicity assessment of UV filters [19, 20], including OMC [21, 22]. The structural knowledge gained in this investigation can help understand OMC-induced developmental toxicity in the early life stages of zebrafish and how this relates to the regulation of the HPT axis. The toxicological data obtained will help inform safety considerations regarding the inclusion of OMC in PCPs and improve human health.

Text S5. Applied methodology to docking simulations.

To *in silico* analysis, proteins and ligands were previously prepared by removed water molecules, merged non-polar hydrogens atoms, and added Gasteiger partial charges using Autodock Tools v1.5.6 and UCSF Chimera 1.15 software's. This automatic protonation simulated an approximate physiological pH of 7.0 to 7.4. No solvent was used; therefore, the structure optimisation and docking simulations were performed in vacuum. The Ramachandran plot generated (RamPlot webserver, https://www.ramplot.in/) confirms the three-dimensional arrangement of the proteins for molecular docking simulations [23] (**Figures S2-S12**). Before carrying out the simulations, the ligand binding sites (pockets) were predicted using the PrankWeb online web server, https://prankweb.cz/ [24], an interactive interface to the P2RANK method [25]. This intuitive tool allows immediate visual analysis of the 3D structure of the protein, the sequence and the list of binding pockets (PDB or AlphaFold) [26]. The grid box constructed for all proteins had the dimensions 30×30×30Å along x, y, and z, considering a grid spacing of 0.375 Å. Molecular docking was validated for root mean square deviation values <2Å, and AutoDock Vina confirmed the results [27, 28]. Docking calculations were performed using the Lamarckian Genetic algorithm approach, and the remaining parameters were set as default values. The estimated binding complex with the lowest Gibbs free energy (ΔG, expressed in kcal/mol) from 10 hybrid runs was analysed for each simulation. The inhibition constant k_i (k_i=exp (−ΔG/RT), expressed in μmol/L) was also predicted in the molecular docking analysis for a better interpretation of the interaction effectiveness. The k_i is a semi-empirical free energy constant usually indicates the receptor inhibition, which virtually explores the dose concentration [29]. The k_i calculation was obtained considering each ΔG, R as the gas constant (1.985×10^{-3} kcal/mol^{-1} K^{-1}) and T (temperature = 298.15 K). The k_i will be lesser if ΔG is negative, indicating greater affinity of the inhibitor for the receptor [30, 31]. To investigate the interactions (amino acid residues, H-bonds, and hydrophobic environment) between drugs and the HPT-axis-related proteins, the UCSF Chimera 1.15 was used. Discovery Studio Visualizer software (BIOVIA, v. 21.1.0.20298) was applied to visualize atomic interactions. All proteins used from *Homo sapiens* and *Danio rerio* were retrieved from AlphaFold Protein Structure Database (https://alphafold.ebi.ac.uk/) through the Uniprot ID (**Table S4**). The Zebrafish Information Network (ZFIN, https://zfin.org/) was used to confirm that the *D. rerio* chosen proteins corresponded to the human ontologue. Moreover, regions of local similarity between sequences from the two species were analysed by the basic local alignment search tool on UniProt (BLAST, https://www.uniprot.org/align) [32] (**Table S4**).

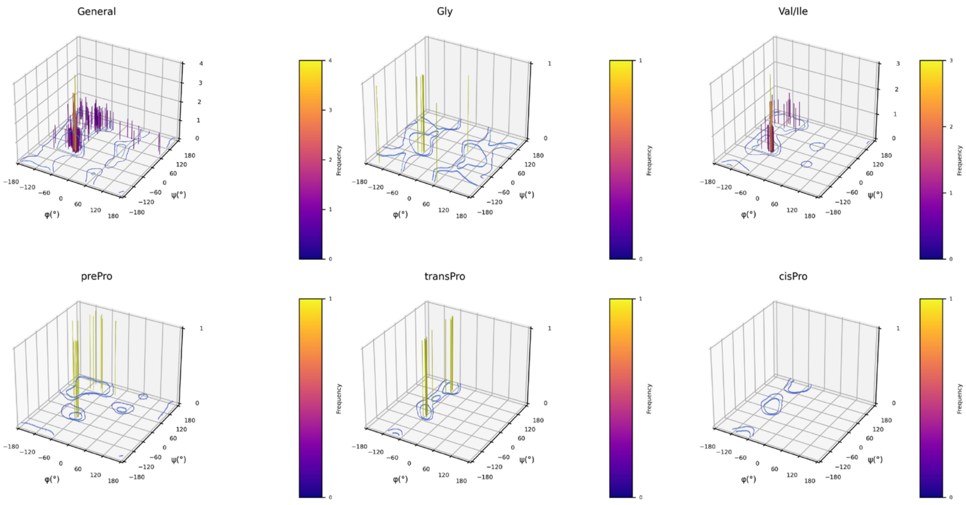

Figure S2. 3D Ramachandran plot from Thyrotropin-releasing hormone receptor (TRHR, human) of six distinct categories: **(a)** general case (Ala and remaining 15 amino acids), **(b)** Gly, **(c)** Val/Ile, **(d)** pre-Pro, **(e)** trans-Pro & **(f)** cis-Pro. Bars represent the frequency of torsion angles.

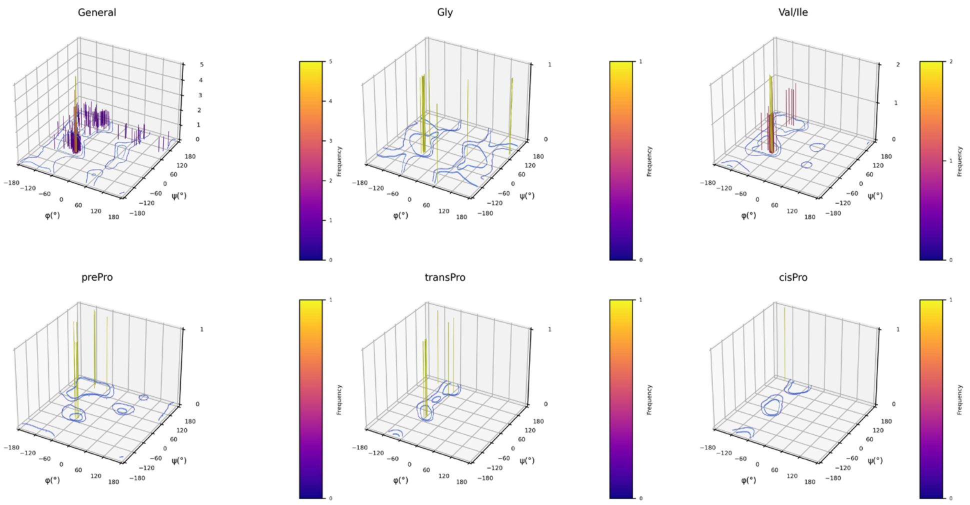

Figure S3. 3D Ramachandran plot from Thyrotropin-releasing hormone receptor (trhrb, zebrafish) of six distinct categories: **(a)** general case (Ala and remaining 15 amino acids), **(b)** Gly, **(c)** Val/Ile, **(d)** pre-Pro, **(e)** trans-Pro & **(f)** cis-Pro. Bars represent the frequency of torsion angles.

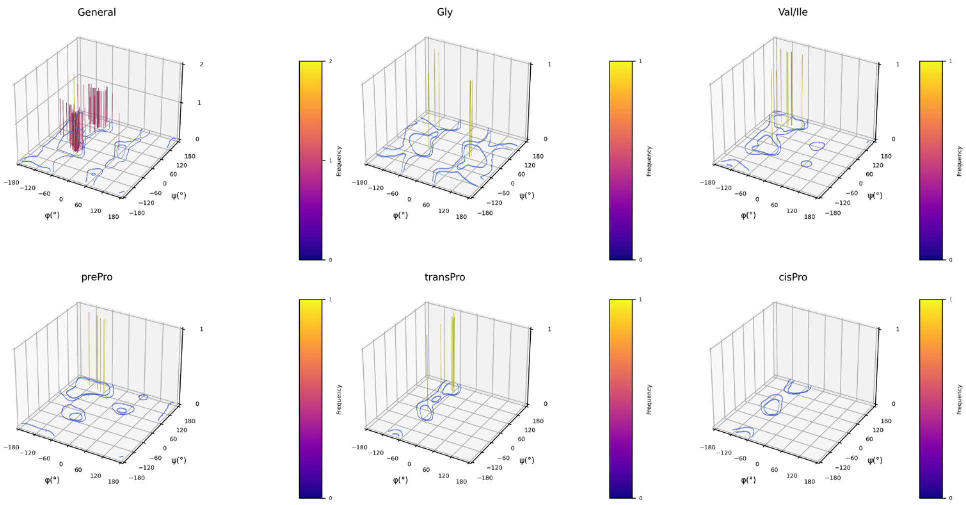

Figure S4. 3D Ramachandran plot from Corticotropin releasing hormone receptor 2 (crhr2, zebrafish) of six distinct categories: **(a)** general case (Ala and remaining 15 amino acids), **(b)** Gly, **(c)** Val/Ile, **(d)** pre-Pro, **(e)** trans-Pro & **(f)** cis-Pro. Bars represent the frequency of torsion angles.

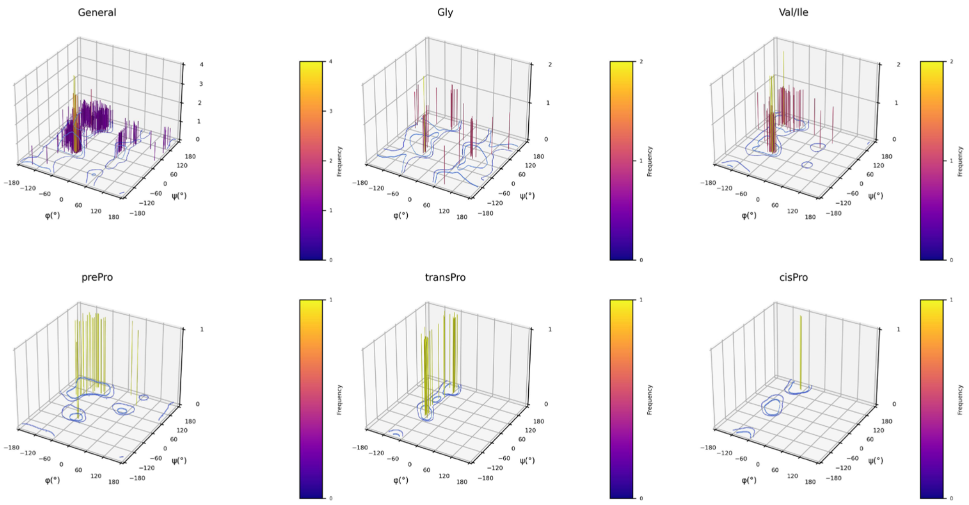

Figure S5. 3D Ramachandran plot from Thyrotropin# receptor (TSHR, humans) of six distinct categories: **(a)** general case (Ala and remaining 15 amino acids), **(b)** Gly, **(c)** Val/Ile, **(d)** pre-Pro, **(e)** trans-Pro & **(f)** cis-Pro. Bars represent the frequency of torsion angles.

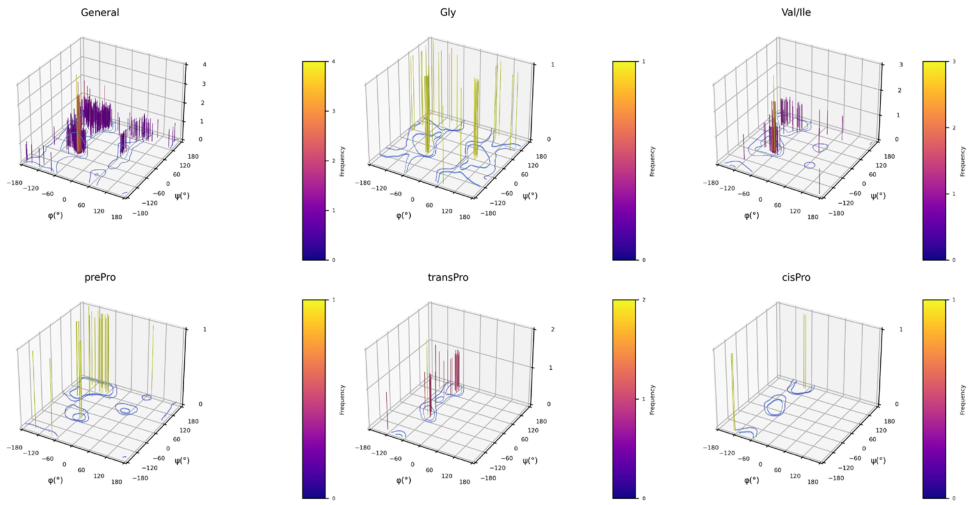

Figure S6. 3D Ramachandran plot from Thyrotropin# receptor (tshr, zebrafish) of six distinct categories: **(a)** general case (Ala and remaining 15 amino acids), **(b)** Gly, **(c)** Val/Ile, **(d)** pre-Pro, **(e)** trans-Pro & **(f)** cis-Pro. Bars represent the frequency of torsion angles.

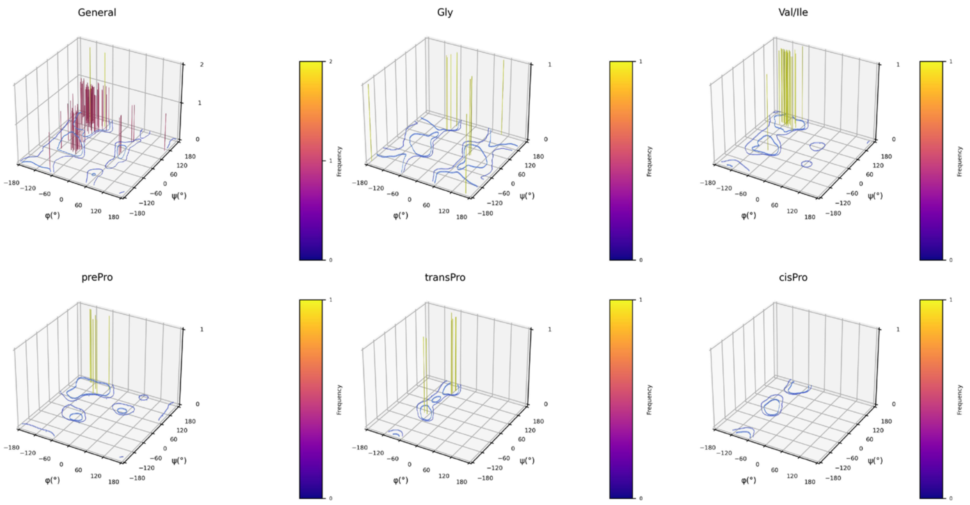

Figure S7. 3D Ramachandran plot from Transthyretin (TTR, humans) of six distinct categories: **(a)** general case (Ala and remaining 15 amino acids), **(b)** Gly, **(c)** Val/Ile, **(d)** pre-Pro, **(e)** trans-Pro & **(f)** cis-Pro. Bars represent the frequency of torsion angles.

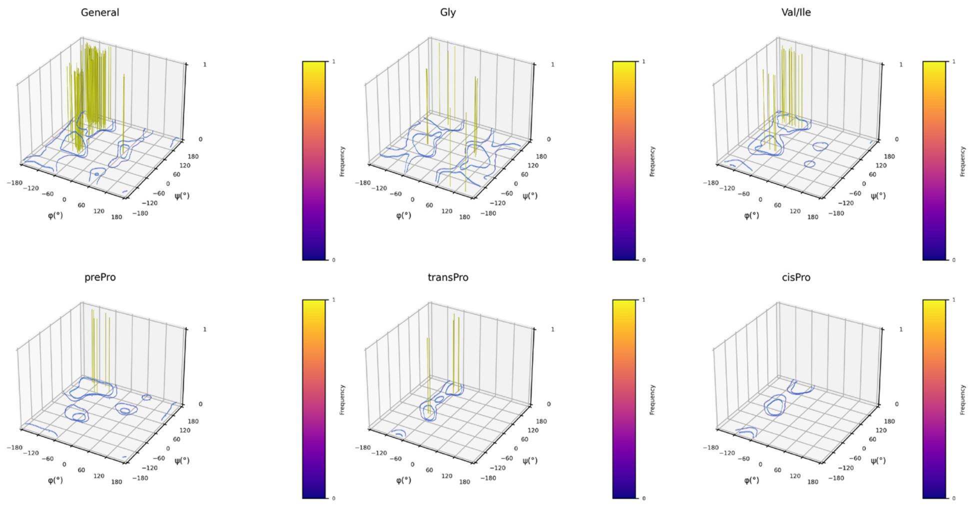

Figure S8. 3D Ramachandran plot from Transthyretin (ttr, zebrafish) of six distinct categories: **(a)** general case (Ala and remaining 15 amino acids), **(b)** Gly, **(c)** Val/Ile, **(d)** pre-Pro, **(e)** trans-Pro & **(f)** cis-Pro. Bars represent the frequency of torsion angles.

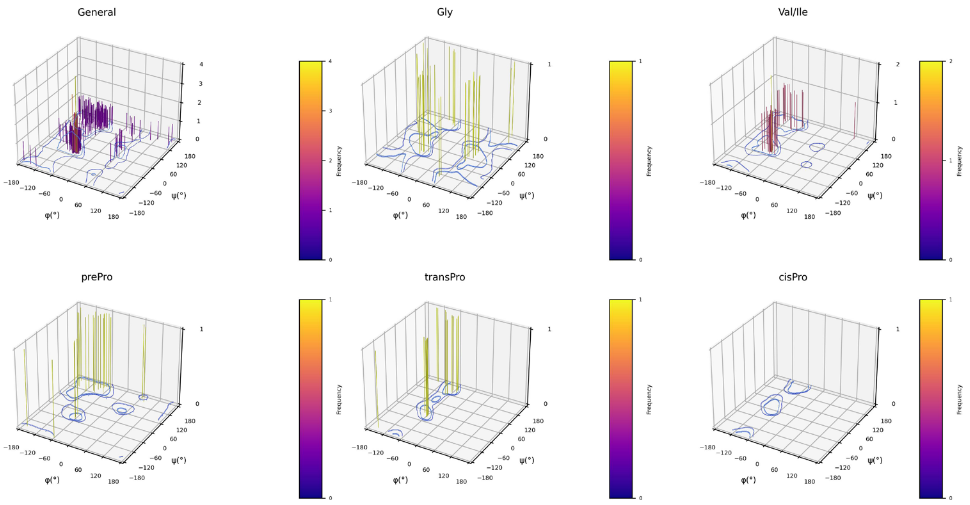

Figure S9. 3D Ramachandran plot from Thyroid hormone receptor alpha (THRA, humans) of six distinct categories: **(a)** general case (Ala and remaining 15 amino acids), **(b)** Gly, **(c)** Val/Ile, **(d)** pre-Pro, **(e)** trans-Pro & **(f)** cis-Pro. Bars represent the frequency of torsion angles.

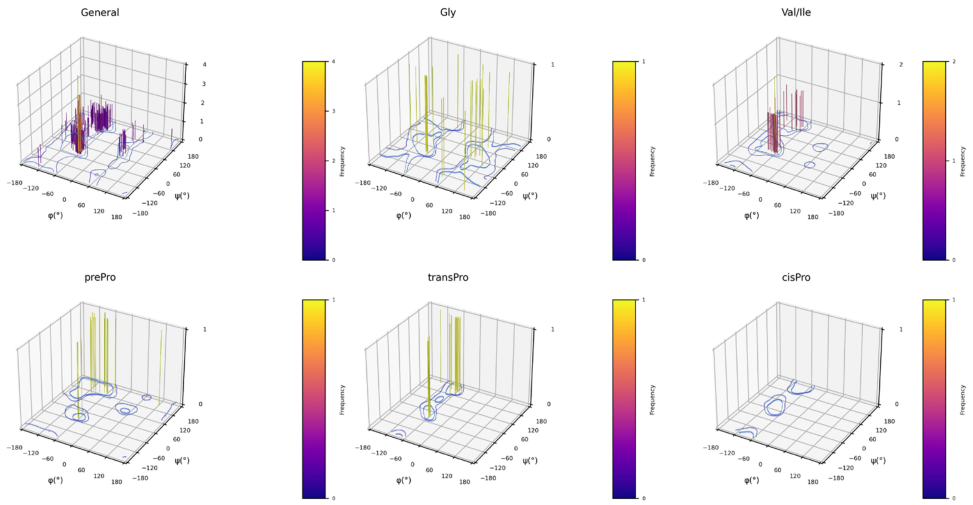

Figure S10. 3D Ramachandran plot from Thyroid hormone receptor alpha-A (thraa, zebrafish) of six distinct categories: **(a)** general case (Ala and remaining 15 amino acids), **(b)** Gly, **(c)** Val/Ile, **(d)** pre-Pro, **(e)** trans-Pro & **(f)** cis-Pro. Bars represent the frequency of torsion angles.

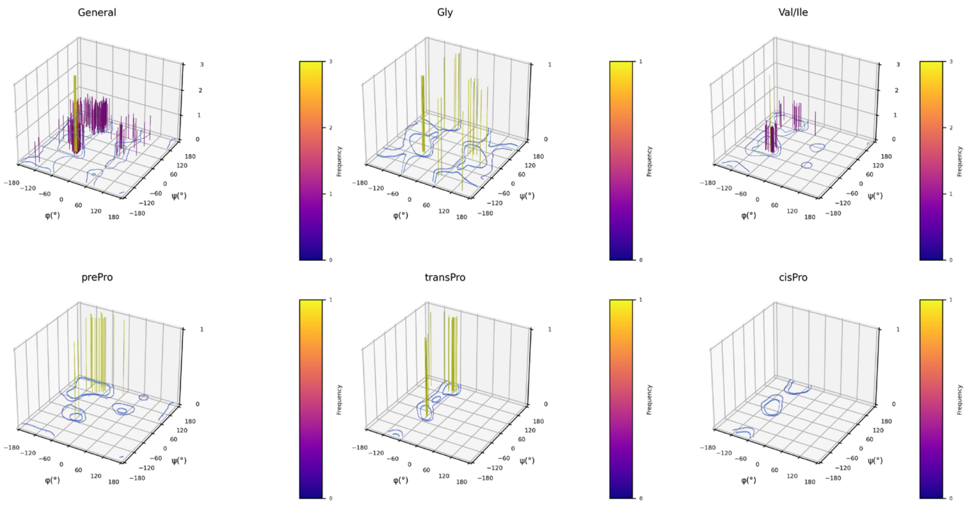

Figure S11. 3D Ramachandran plot from Thyroid hormone receptor beta (THRB, humans) of six distinct categories: **(a)** general case (Ala and remaining 15 amino acids), **(b)** Gly, **(c)** Val/Ile, **(d)** pre-Pro, **(e)** trans-Pro & **(f)** cis-Pro. Bars represent the frequency of torsion angles.

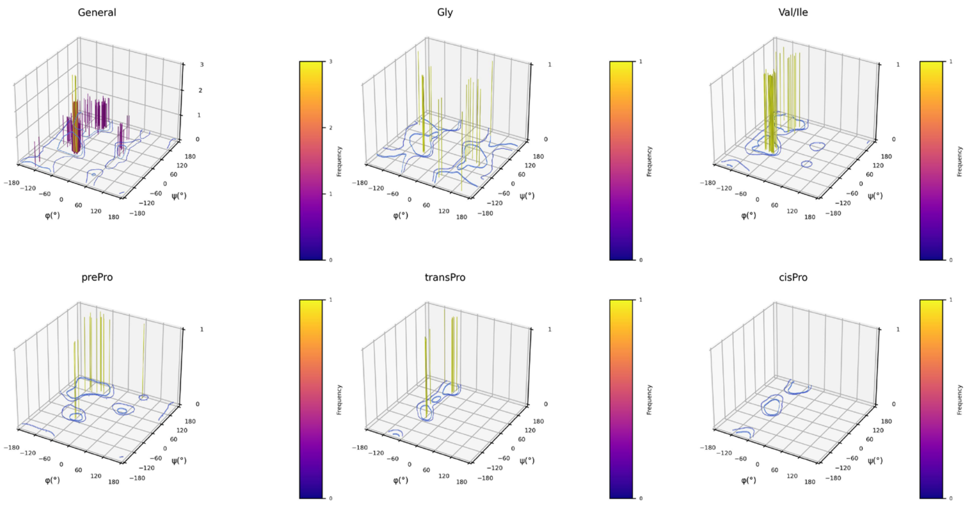

Figure S12. 3D Ramachandran plot from Thyroid hormone receptor beta (thrb, zebrafish) of six distinct categories: **(a)** general case (Ala and remaining 15 amino acids), **(b)** Gly, **(c)** Val/Ile, **(d)** pre-Pro, **(e)** trans-Pro & **(f)** cis-Pro. Bars represent the frequency of torsion angles.

Table S4. UniProt / AlphaFold (AF) Accession, Protein and Gene names, % of identity between the species analysed and organism test. Legend: # Thyrotropin is also designated of thyroid stimulating hormone (TSH); N/A: not applicable.

UniProt /AF ID Accession	Protein	Gene	% Identity	Organism
P34981	Thyrotropin-releasing hormone receptor	*TRHR*	65.39%	*H. sapiens (Human)*
A8E7L5		*trhrb*		*D. rerio (Zebrafish)*
A8WG03	Corticotropin releasing hormone receptor 2	*crhr2*	N/A	*D. rerio (Zebrafish)*
P16473	Thyrotropin# receptor	*TSHR*	58.93%	*H. sapiens (Human)*
C0SKM5		*tshr*		*D. rerio (Zebrafish)*
P02766	Transthyretin	*TTR*	48.63%	*H. sapiens (Human)*
B8JLL8		*ttr*		*D. rerio (Zebrafish)*
P10827	Thyroid hormone receptor alpha	*THRA*	75.43%	*H. sapiens (Human)*
Q98867	Thyroid hormone receptor alpha-A	*thraa*		*D. rerio (Zebrafish)*
P10828	Thyroid hormone receptor beta	*THRB*	87.05%	*H. sapiens (Human)*
Q9PVE4		*thrb*		*D. rerio (Zebrafish)*

Table S5. Nomenclature, ligand and PubChem IDs, and Chemical Abstracts Service Registry Number (CASRN) of ligands.

S. no	Nomenclature	Ligand ID	PubChem ID	CASRN
1	Triiodothyronine	T3	5920	6893-02-3
2	Propylthiouracil	3CJ	657298	51-52-5
3	Octylmethoxycinnamate	OMC	5355130	5466-77-3

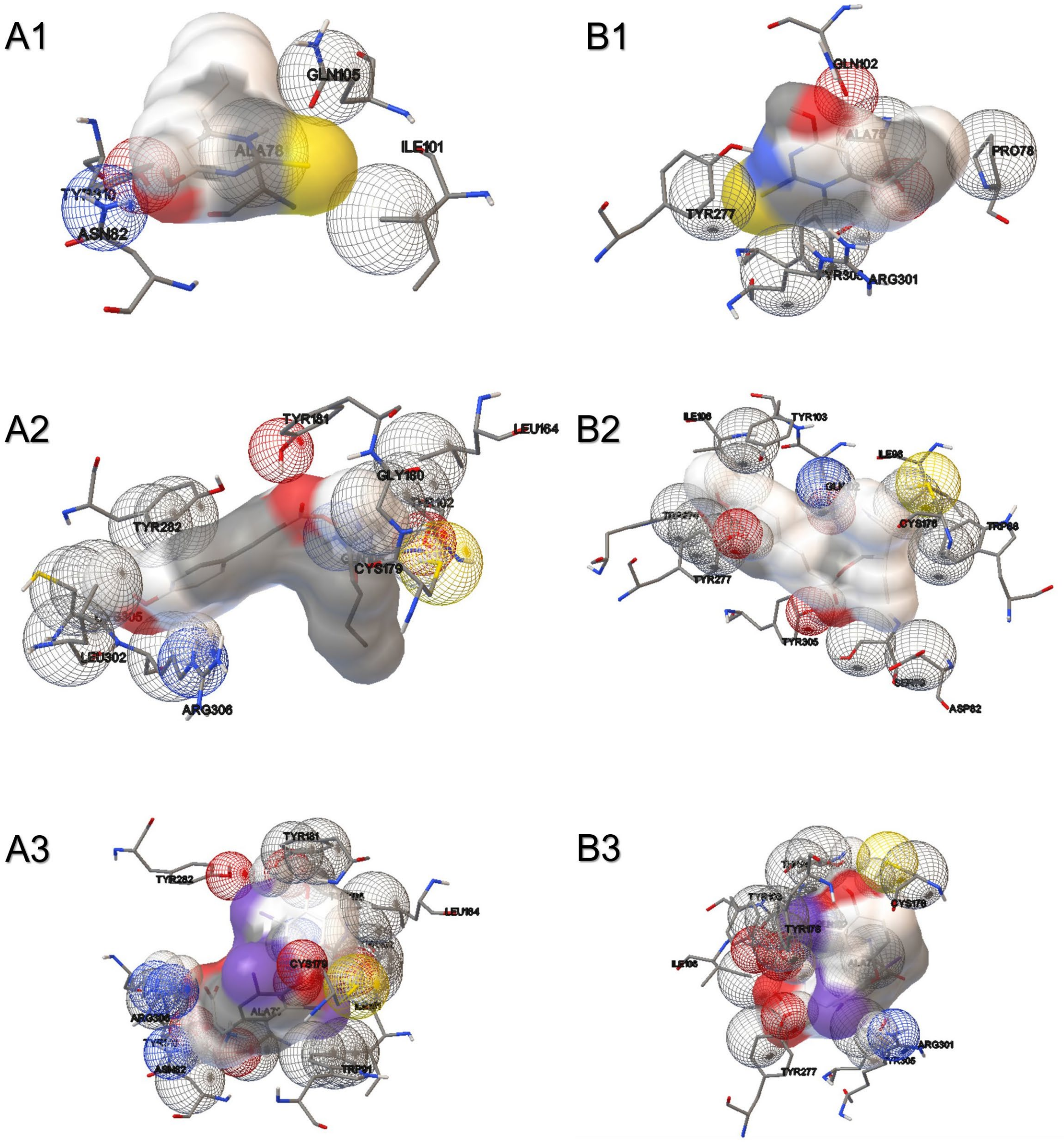

Figure S13. 3D-representation of preferred conformation and interactions with amino acid residues of the complex between the ligands propylthiouracil (PTU, blue), octylmethoxycinnamate (OMC, orange) and triiodothyronine (T3, grey) with Thyrotropin-Releasing Hormone Receptor from **(A)** humans and **(B)** zebrafish, using Autodock.

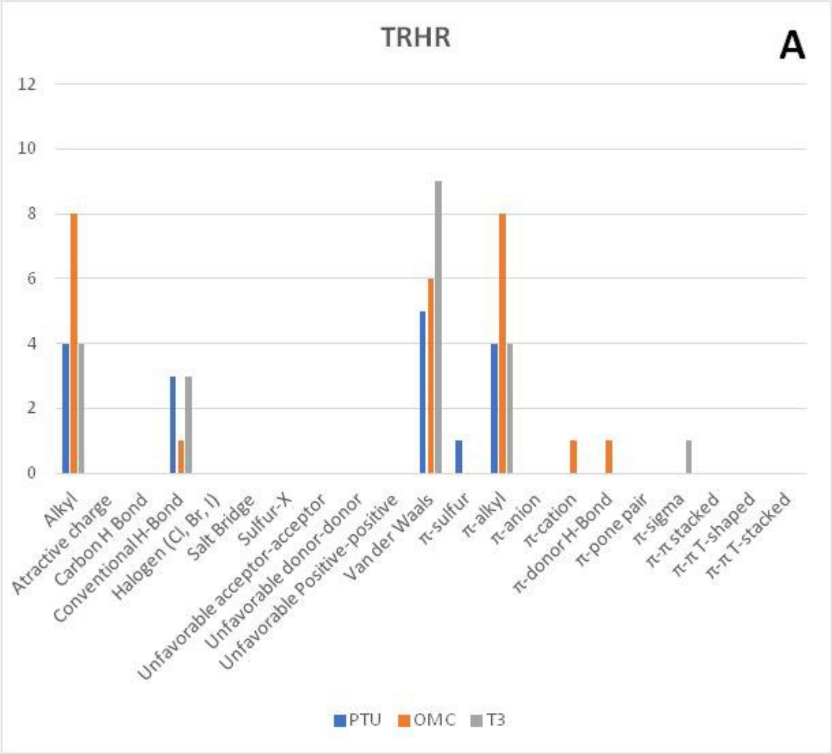

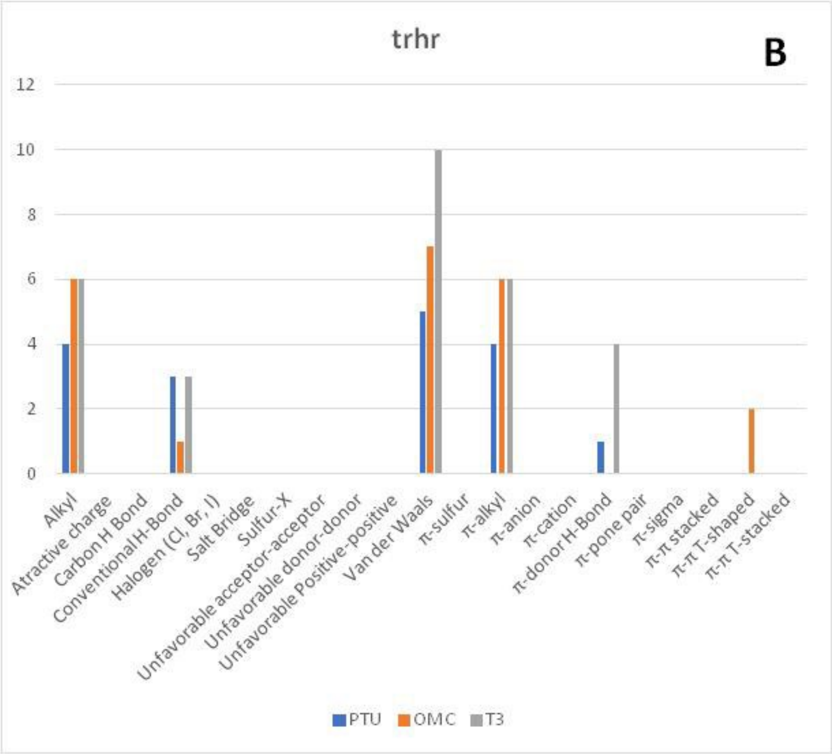

Figure S14. Atomic interactions type by Discovery Studio of the complex between the ligands propylthiouracil (PTU, blue), octylmethoxycinnamate (OMC, orange) and triiodothyronine (T3, grey) with Thyrotropin-Releasing Hormone Receptor from **(A)** humans and **(B)** zebrafish.

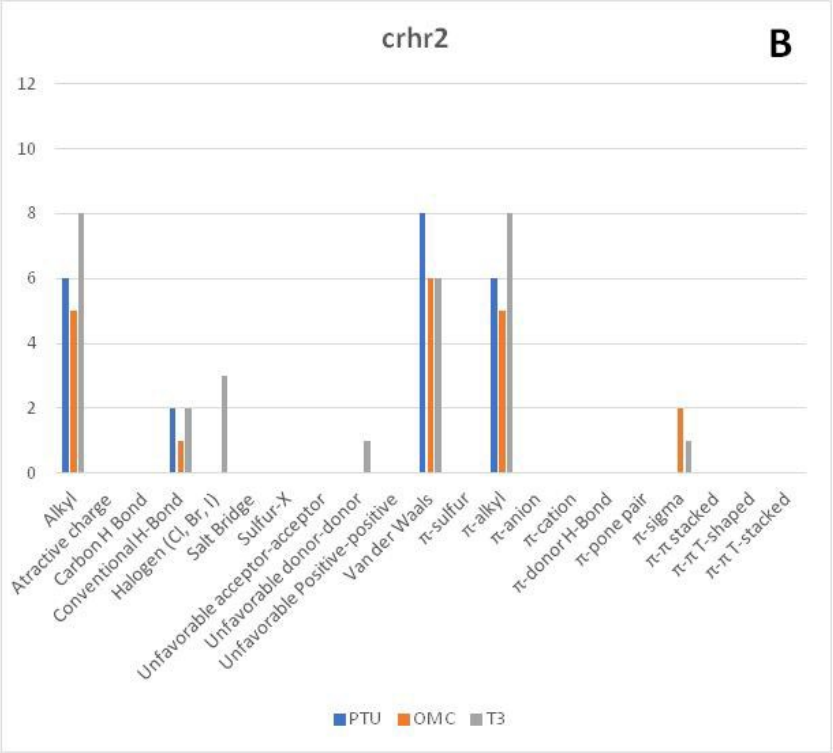

Figure S15. Atomic interactions type by Discovery Studio of the complex between the ligands propylthiouracil (PTU, blue), octylmethoxycinnamate (OMC, orange) and triiodothyronine (T3, grey) with Corticotropin-Releasing Hormone Receptor from **(B)** zebrafish.

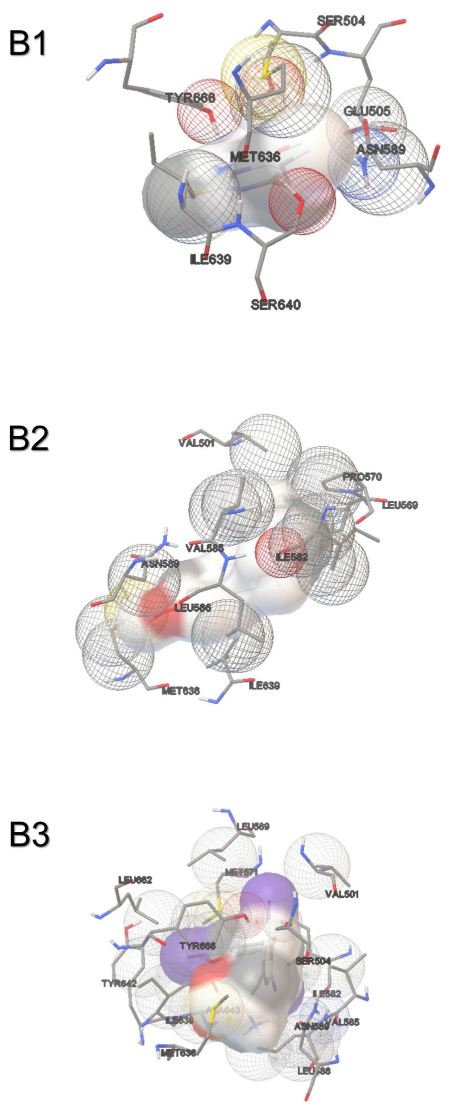

Figure S16. 3D-representation of preferred conformation and interactions with amino acid residues of the complex between the ligands propylthiouracil (PTU, blue), octylmethoxycinnamate (OMC, orange) and triiodothyronine (T3, grey) with Thyroid Stimulating Hormone Receptor (or Thyrotropin receptor) from **(B)** zebrafish, using Autodock.

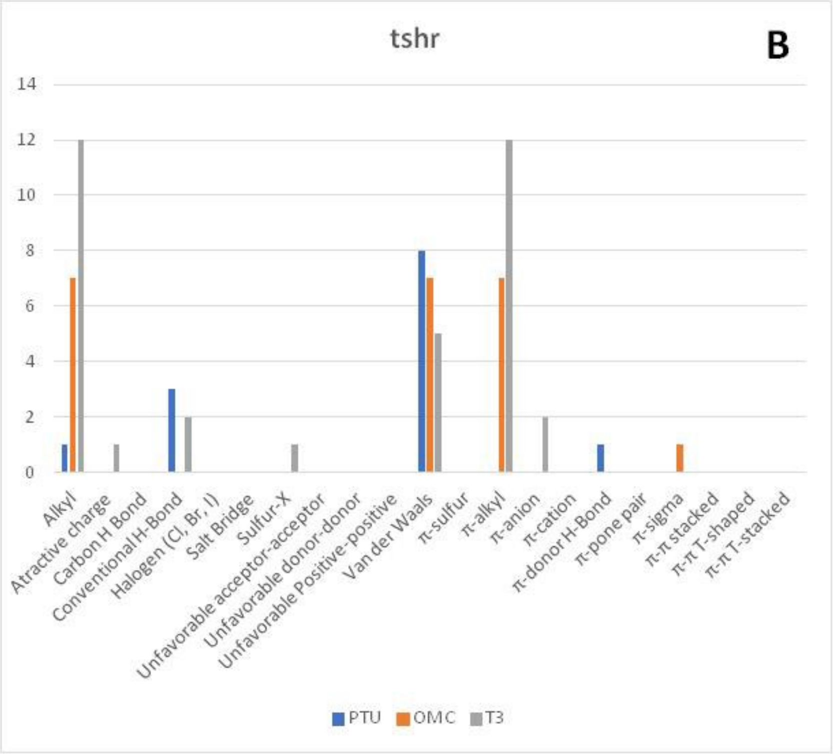

Figure S17. Atomic interactions type by Discovery Studio of the complex between the ligands propylthiouracil (PTU, blue), octylmethoxycinnamate (OMC, orange) and triiodothyronine (T3, grey) with Thyroid Stimulating Hormone Receptor (or Thyrotropin receptor) from **(B)** zebrafish.

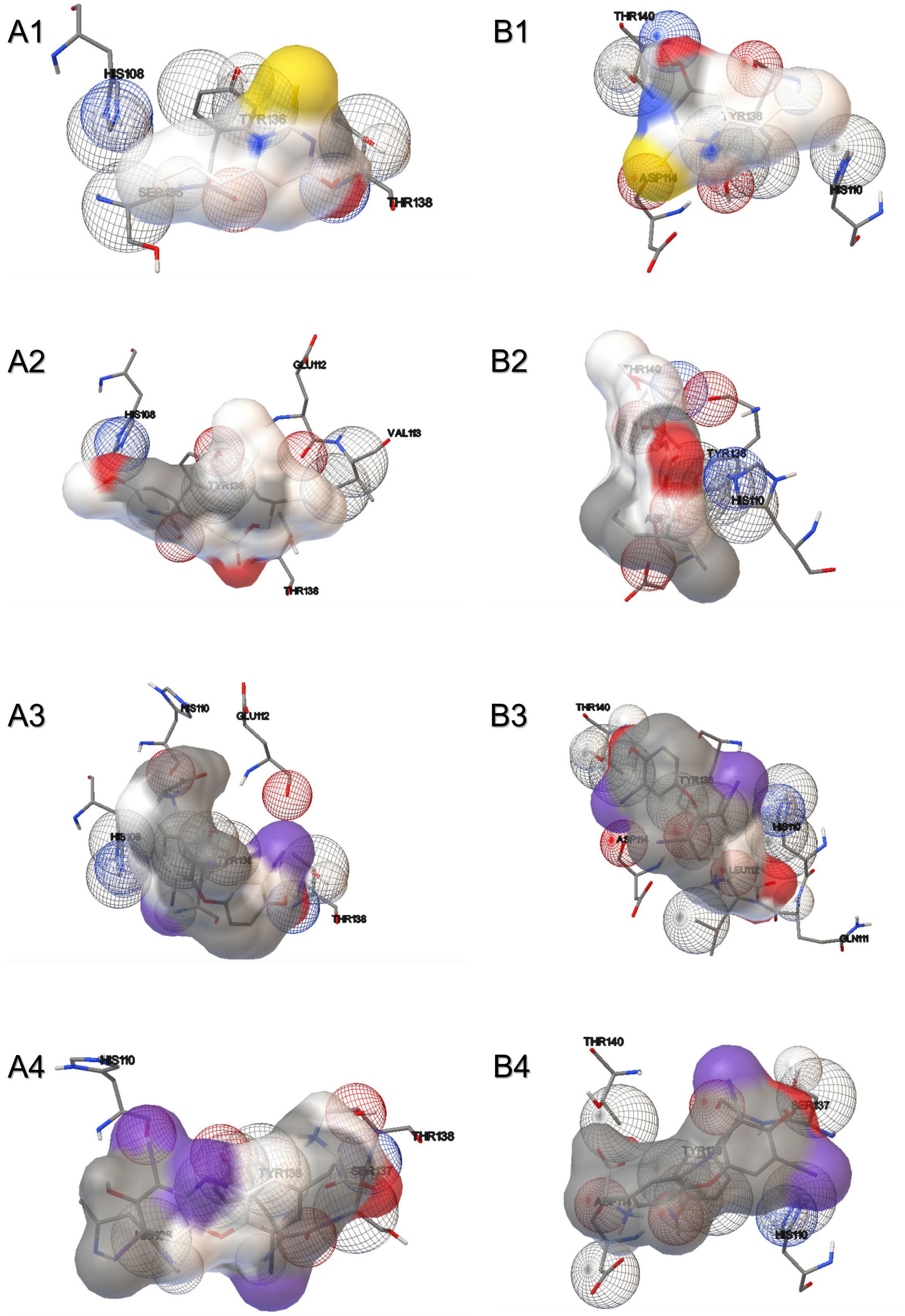

Figure S18. 3D-representation of preferred conformation and interactions with amino acid residues of the complex between the ligands propylthiouracil (PTU, blue), octylmethoxycinnamate (OMC, orange) and triiodothyronine (T3, grey) with Transthyretin from **(A)** humans and **(B)** zebrafish, using Autodock.

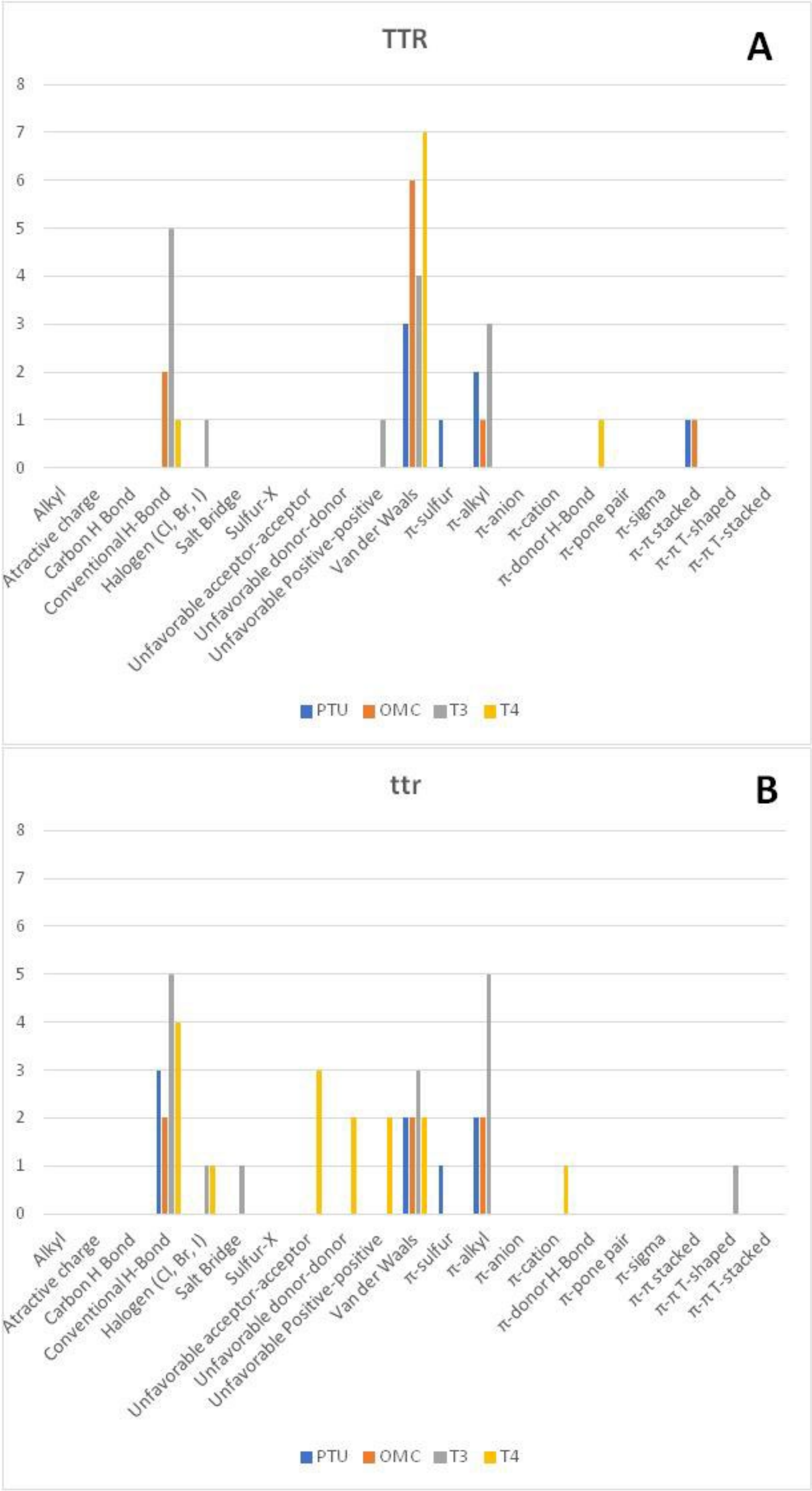

Figure S19. Atomic interactions type by Discovery Studio of the complex between the ligands propylthiouracil (PTU, blue), octylmethoxycinnamate (OMC, orange), triiodothyronine (T3, grey) and tetraiodothyronine (T4, yellow) with Transthyretin from **(A)** humans and **(B)** zebrafish.

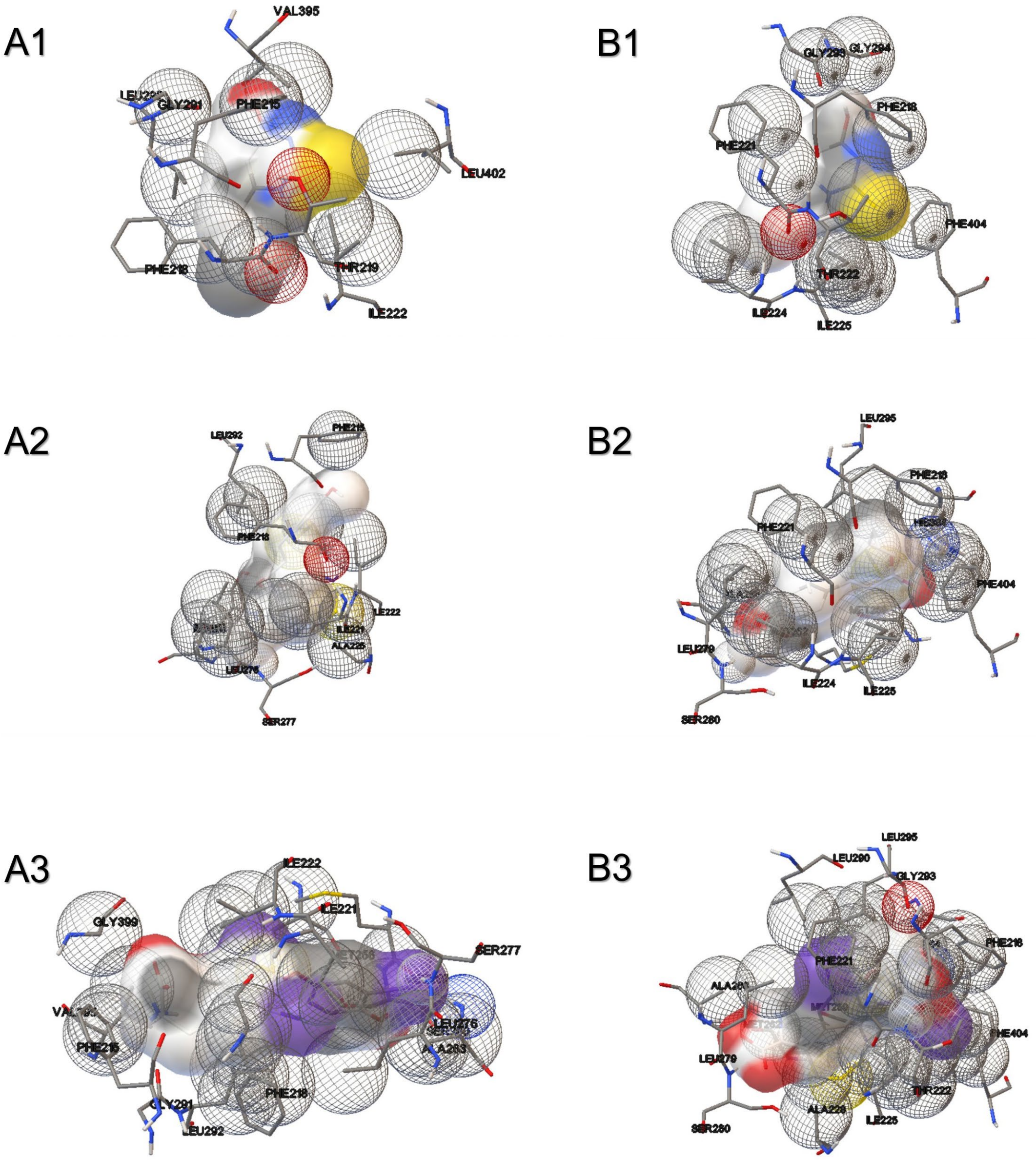

Figure S20. 3D-representation of preferred conformation and interactions with amino acid residues of the complex between the ligands propylthiouracil (PTU, blue), octylmethoxycinnamate (OMC, orange) and triiodothyronine (T3, grey) with Thyroid Hormone Receptor alpha from **(A)** humans and **(B)** zebrafish, using Autodock.

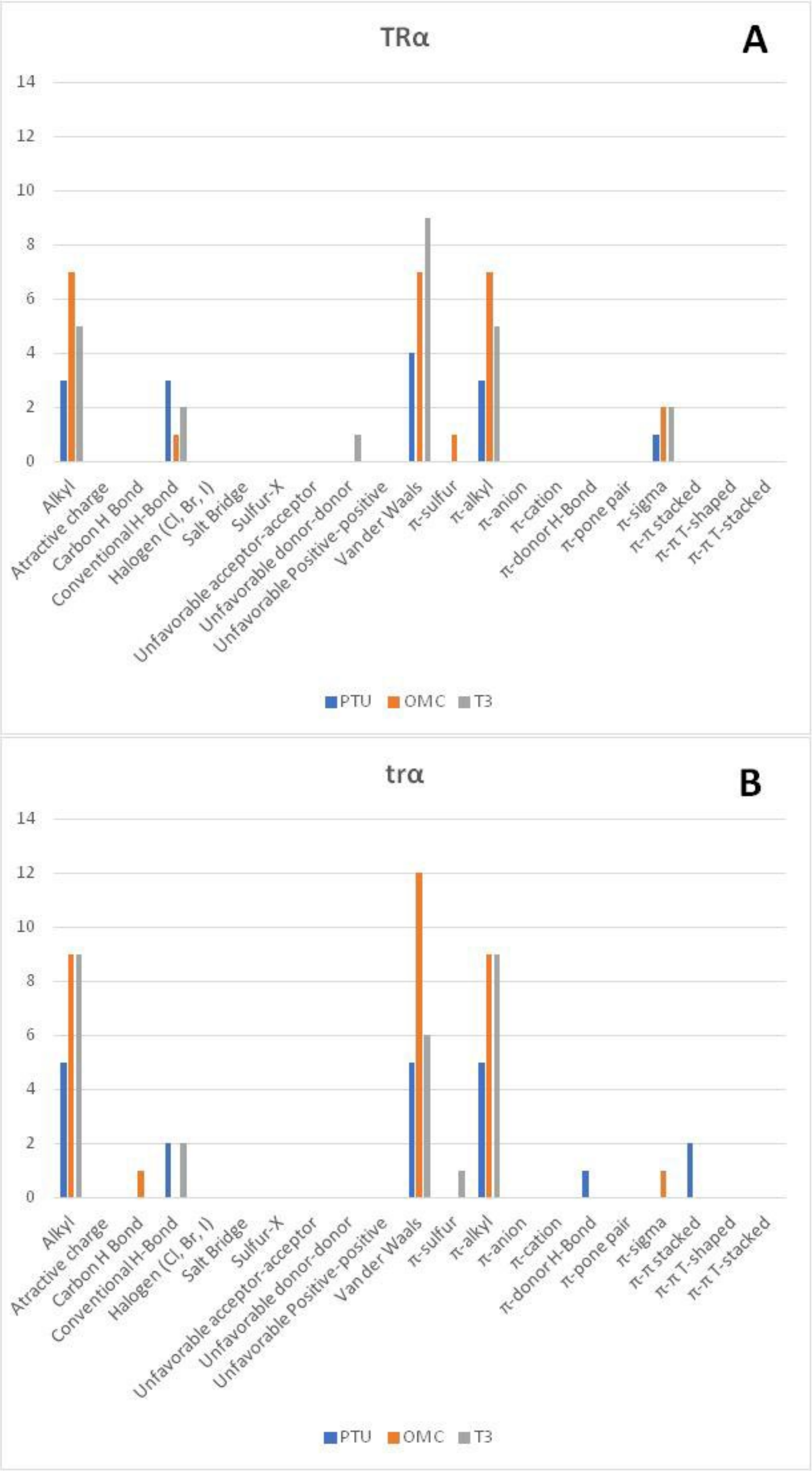

Figure S21. Atomic interactions type by Discovery Studio of the complex between the ligands propylthiouracil (PTU, blue), octylmethoxycinnamate (OMC, orange) and triiodothyronine (T3, grey) with Thyroid Hormone Receptor alpha from **(A)** humans and **(B)** zebrafish.

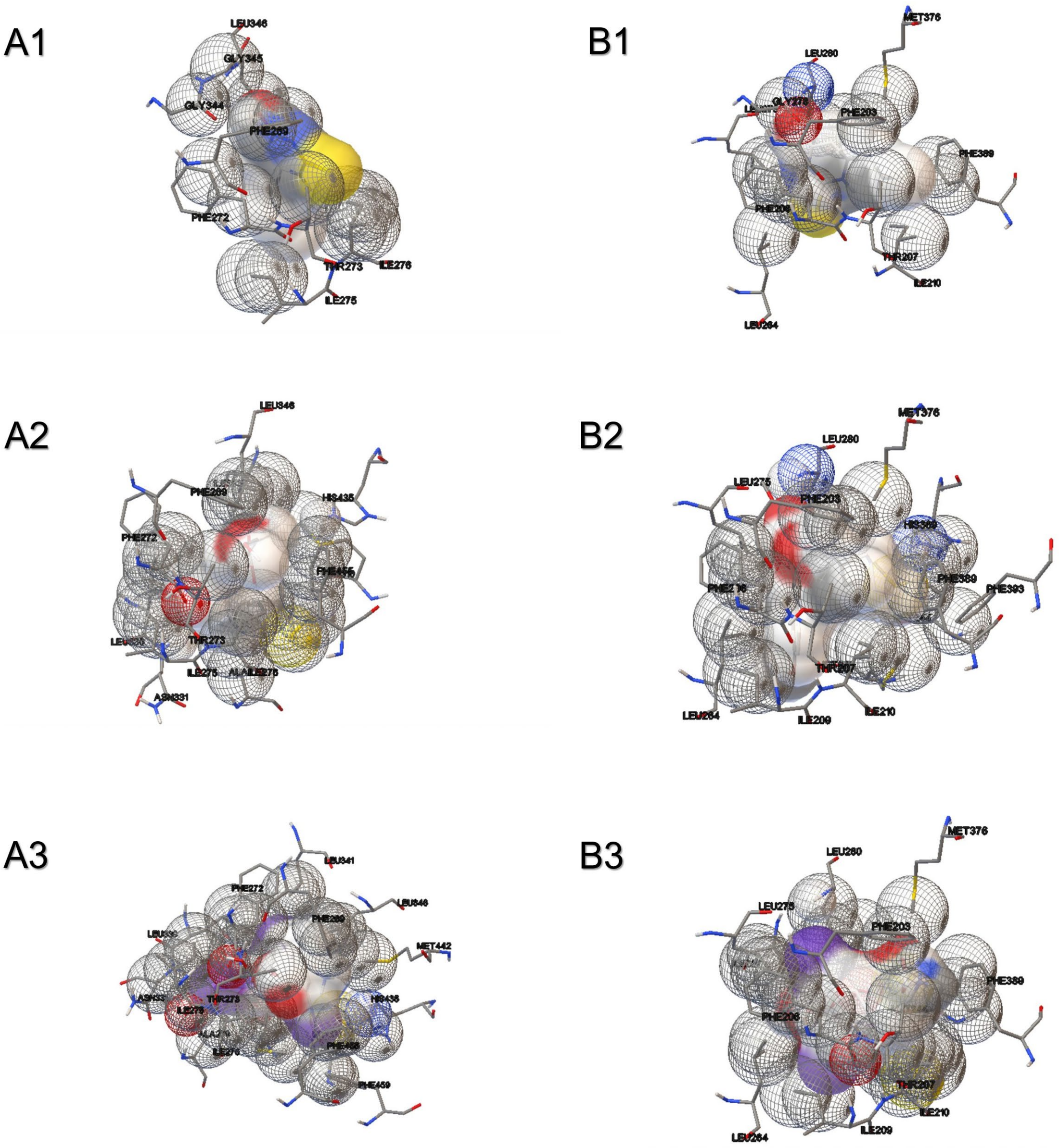

Figure S22. 3D-representation of preferred conformation and interactions with amino acid residues of the complex between the ligands propylthiouracil (PTU, blue), octylmethoxycinnamate (OMC, orange) and triiodothyronine (T3, grey) with Thyroid Hormone Receptor beta from **(A)** humans and **(B)** zebrafish, using Autodock.

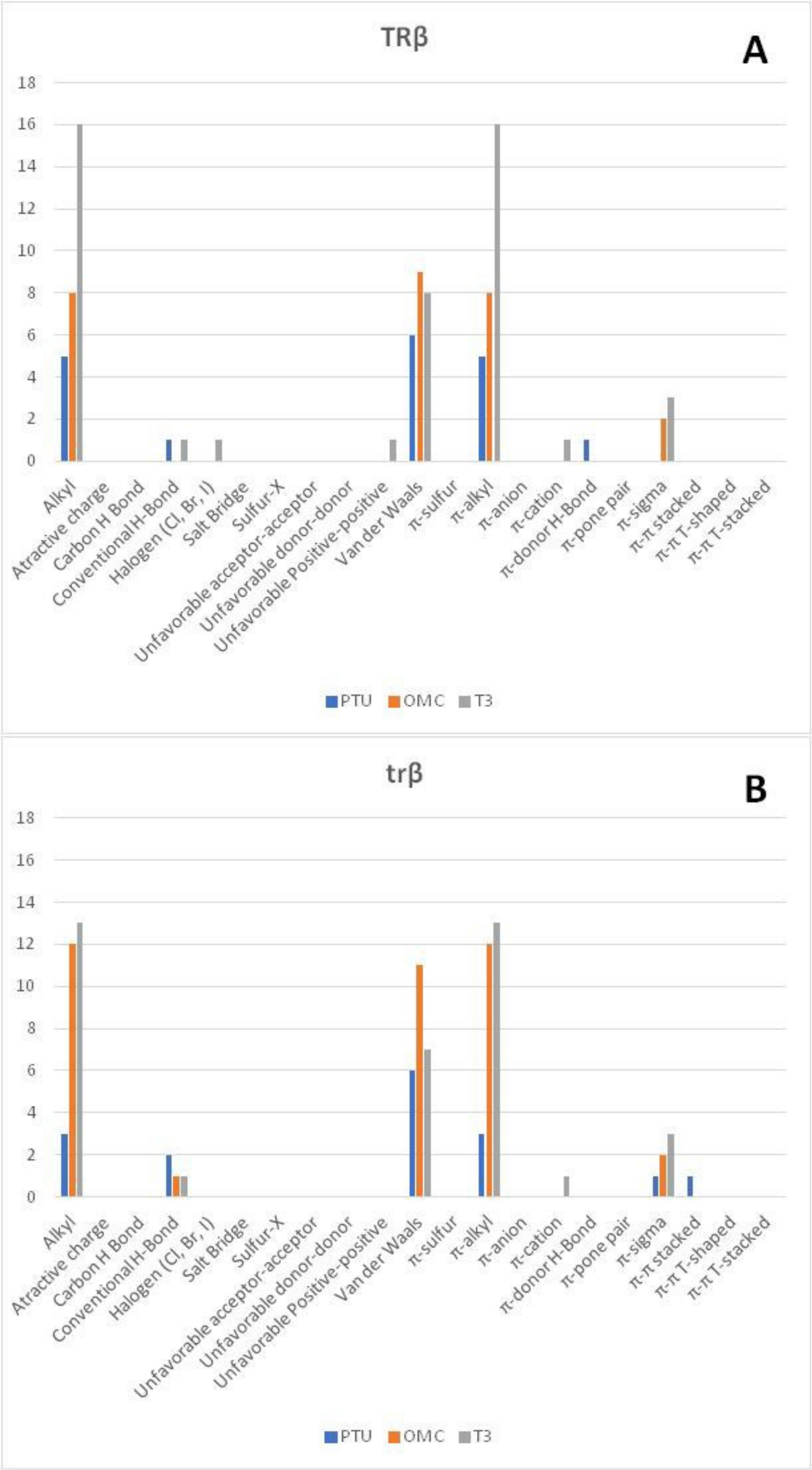

Figure S23. Atomic interactions type by Discovery Studio of the complex between the ligands propylthiouracil (PTU, blue), octylmethoxycinnamate (OMC, orange) and triiodothyronine (T3, grey) with Thyroid Hormone Receptor beta from **(A)** humans and **(B)** zebrafish.

REFERENCES

1. Lipinski, C. A., F. Lombardo, B. W. Dominy and P. J. Feeney. "Experimental and computational approaches to estimate solubility and permeability in drug discovery and development settings." *Adv Drug Deliv Rev* 46 (**2001**): 3-26. 10.1016/s0169-409x(00)00129-0. https://www.ncbi.nlm.nih.gov/pubmed/11259830.

2. Kuhne, R., K. Hilscherova, M. Smutna, F. Lessmollmann and G. Schuurmann. "In silico bioavailability triggers applied to direct and indirect thyroid hormone disruptors." *Chemosphere* 348 (**2024**): 140611. 10.1016/j.chemosphere.2023.140611. https://www.ncbi.nlm.nih.gov/pubmed/37972869.

3. Varshavsky, J., A. Smith, A. Wang, E. Hom, M. Izano, H. Huang, A. Padula and T. J. Woodruff. "Heightened susceptibility: A review of how pregnancy and chemical exposures influence maternal health." *Reprod Toxicol* 92 (**2020**): 14-56. 10.1016/j.reprotox.2019.04.004. https://www.ncbi.nlm.nih.gov/pubmed/31055053.

4. Maipas, S. and P. Nicolopoulou-Stamati. "Sun lotion chemicals as endocrine disruptors." *Hormones (Athens)* 14 (**2015**): 32-46. 10.1007/BF03401379. https://www.ncbi.nlm.nih.gov/pubmed/25885102.

5. Krause, M., A. Klit, M. Blomberg Jensen, T. Soeborg, H. Frederiksen, M. Schlumpf, W. Lichtensteiger, N. E. Skakkebaek and K. T. Drzewiecki. "Sunscreens: Are they beneficial for health? An overview of endocrine disrupting properties of uv-filters." *Int J Androl* 35 (**2012**): 424-36. 10.1111/j.1365-2605.2012.01280.x. https://www.ncbi.nlm.nih.gov/pubmed/22612478.

6. Janjua, N. R., B. Kongshoj, A. M. Andersson and H. C. Wulf. "Sunscreens in human plasma and urine after repeated whole-body topical application." *J Eur Acad Dermatol Venereol* 22 (**2008**): 456-61. 10.1111/j.1468-3083.2007.02492.x. https://www.ncbi.nlm.nih.gov/pubmed/18221342.

7. Matta, M. K., J. Florian, R. Zusterzeel, N. R. Pilli, V. Patel, D. A. Volpe, Y. Yang, L. Oh, E. Bashaw, I. Zineh, *et al.* "Effect of sunscreen application on plasma concentration of sunscreen active ingredients: A randomized clinical trial." *JAMA* 323 (**2020**): 256-67. 10.1001/jama.2019.20747. https://www.ncbi.nlm.nih.gov/pubmed/31961417.

8. Axelstad, M., J. Boberg, K. S. Hougaard, S. Christiansen, P. R. Jacobsen, K. R. Mandrup, C. Nellemann, S. P. Lund and U. Hass. "Effects of pre- and postnatal exposure to the uv-filter octyl methoxycinnamate (omc) on the reproductive, auditory and neurological development of rat offspring." *Toxicol Appl Pharmacol* 250 (**2011**): 278-90. 10.1016/j.taap.2010.10.031. https://www.ncbi.nlm.nih.gov/pubmed/21059369.

9. Szwarcfarb, B., S. Carbone, R. Reynoso, G. Bollero, O. Ponzo, J. Moguilevsky and P. Scacchi. "Octyl-methoxycinnamate (omc), an ultraviolet (uv) filter, alters lhrh and amino acid neurotransmitters release from hypothalamus of immature rats." *Exp Clin Endocrinol Diabetes* 116 (**2008**): 94-8. 10.1055/s-2007-1004589. https://www.ncbi.nlm.nih.gov/pubmed/18286425.

10. Carbone, S., B. Szwarcfarb, R. Reynoso, O. J. Ponzo, N. Cardoso, E. Ale, J. A. Moguilevsky and P. Scacchi. "In vitro effect of octyl - methoxycinnamate (omc) on the release of gn-rh and amino acid neurotransmitters by hypothalamus of adult rats." *Exp Clin Endocrinol Diabetes* 118 (**2010**): 298-303. 10.1055/s-0029-1224153. https://www.ncbi.nlm.nih.gov/pubmed/20198561.

11. Lichtensteiger, W., C. Bassetti-Gaille, O. Faass, M. Axelstad, J. Boberg, S. Christiansen, H. Rehrauer, J. K. Georgijevic, U. Hass, A. Kortenkamp, *et al.* "Differential gene expression patterns in developing sexually dimorphic rat brain regions exposed to antiandrogenic, estrogenic, or complex endocrine disruptor mixtures: Glutamatergic synapses as target." *Endocrinology* 156 (**2015**): 1477-93. 10.1210/en.2014-1504. https://www.ncbi.nlm.nih.gov/pubmed/25607892.

12. Vela-Soria, F., M. E. Gallardo-Torres, O. Ballesteros, C. Diaz, J. Perez, A. Navalon, M. F. Fernandez and N. Olea. "Assessment of parabens and ultraviolet filters in human placenta tissue by ultrasound-assisted extraction and ultra-high performance liquid chromatography-tandem mass spectrometry." *J Chromatogr A* 1487 (**2017**): 153-61. 10.1016/j.chroma.2017.01.041. https://www.ncbi.nlm.nih.gov/pubmed/28129936.

13. Schlumpf, M., K. Kypke, C. C. Vöt, M. Birchler, S. Durrer, O. Faass, C. Ehnes, M. Fuetsch, C. Gaille, M. Henseler, *et al.* "Endocrine active uv filters:: Developmental toxicity and exposure through breast milk." *Chimia* 62 (**2008**): 345-51. 10.2533/chimia.2008.345.

14. Schlumpf, M., K. Kypke, M. Wittassek, J. Angerer, H. Mascher, D. Mascher, C. Vokt, M. Birchler and W. Lichtensteiger. "Exposure patterns of uv filters, fragrances, parabens, phthalates, organochlor pesticides, pbdes, and pcbs in human milk: Correlation of uv filters with use of cosmetics." *Chemosphere* 81 (**2010**): 1171-83. 10.1016/j.chemosphere.2010.09.079. https://www.ncbi.nlm.nih.gov/pubmed/21030064.

15. Wang, B., Y. Jin, J. Li, F. Yang, H. Lu, J. Zhou, S. Liu, Z. Shen, X. Yu and T. Yuan. "Exploring environmental obesogenous effects of organic ultraviolet filters on children from a case-control study." *Chemosphere* 341 (**2023**): 139883. 10.1016/j.chemosphere.2023.139883. https://www.ncbi.nlm.nih.gov/pubmed/37672813.

16. Alonso, M. B., M. L. Feo, C. Corcellas, P. Gago-Ferrero, C. P. Bertozzi, J. Marigo, L. Flach, A. C. Meirelles, V. L. Carvalho, A. F. Azevedo, *et al.* "Toxic heritage: Maternal transfer of pyrethroid insecticides and sunscreen agents in dolphins from brazil." *Environ Pollut* 207 (**2015**): 391-402. 10.1016/j.envpol.2015.09.039. https://www.ncbi.nlm.nih.gov/pubmed/26453834.

17. Lorigo, M., C. Quintaneiro, L. Breitenfeld and E. Cairrao. "Exposure to uv-b filter octylmethoxycinnamate and human health effects: Focus on endocrine disruptor actions." *Chemosphere* 358 (**2024**): 142218. 10.1016/j.chemosphere.2024.142218. https://www.ncbi.nlm.nih.gov/pubmed/38704047.

18. Lorigo, M., C. Quintaneiro, L. Breitenfeld and E. Cairrao. "Effects associated with exposure to the emerging contaminant octyl-methoxycinnamate (a uv-b filter) in the aquatic environment: A review." *J Toxicol Environ Health B Crit Rev* 27 (**2024**): 55-72. 10.1080/10937404.2023.2296897. https://www.ncbi.nlm.nih.gov/pubmed/38146151.

19. Kraft, M., L. Golz, M. Rinderknecht, J. Koegst, T. Braunbeck and L. Baumann. "Developmental exposure to triclosan and benzophenone-2 causes morphological alterations in zebrafish (danio rerio) thyroid follicles and eyes." *Environ Sci Pollut Res Int* 30 (**2023**): 33711-24. 10.1007/s11356-022-24531-2. https://www.ncbi.nlm.nih.gov/pubmed/36495432.

20. Quintaneiro, C., B. Teixeira, J. L. Benede, A. Chisvert, A. Soares and M. S. Monteiro. "Toxicity effects of the organic uv-filter 4-methylbenzylidene camphor in zebrafish embryos." *Chemosphere* 218 (**2019**): 273-81. 10.1016/j.chemosphere.2018.11.096. https://www.ncbi.nlm.nih.gov/pubmed/30472611.

21. Chu, S., B. R. Kwon, Y. M. Lee, K. D. Zoh and K. Choi. "Effects of 2-ethylhexyl-4-methoxycinnamate (ehmc) on thyroid hormones and genes associated with thyroid, neurotoxic, and nephrotoxic responses in adult and larval zebrafish (danio rerio)." *Chemosphere* 263 (**2021**): 128176. 10.1016/j.chemosphere.2020.128176. https://www.ncbi.nlm.nih.gov/pubmed/33297144.

22. Ka, Y. and K. Ji. "Waterborne exposure to avobenzone and octinoxate induces thyroid endocrine disruption in wild-type and thralphaa(-/-) zebrafish larvae." *Ecotoxicology* 31 (**2022**): 948-55. 10.1007/s10646-022-02555-1. https://www.ncbi.nlm.nih.gov/pubmed/35622199.

23. Park, S. W., B. H. Lee, S. H. Song and M. K. Kim. "Revisiting the ramachandran plot based on statistical analysis of static and dynamic characteristics of protein structures." *J Struct Biol* 215 (**2023**): 107939. 10.1016/j.jsb.2023.107939. https://www.ncbi.nlm.nih.gov/pubmed/36707040.

24. Jakubec, D., P. Skoda, R. Krivak, M. Novotny and D. Hoksza. "Prankweb 3: Accelerated ligand-binding site predictions for experimental and modelled protein structures." *Nucleic Acids Res* 50 (**2022**): W593-W97. 10.1093/nar/gkac389. https://www.ncbi.nlm.nih.gov/pubmed/35609995.

25. Krivak, R. and D. Hoksza. "P2rank: Machine learning based tool for rapid and accurate prediction of ligand binding sites from protein structure." *J Cheminform* 10 (**2018**): 39. 10.1186/s13321-018-0285-8. https://www.ncbi.nlm.nih.gov/pubmed/30109435.

26. Carpenter, K. A. and R. B. Altman. "Databases of ligand-binding pockets and protein-ligand interactions." *Comput Struct Biotechnol J* 23 (**2024**): 1320-38. 10.1016/j.csbj.2024.03.015. https://www.ncbi.nlm.nih.gov/pubmed/38585646.

27. Eberhardt, J., D. Santos-Martins, A. F. Tillack and S. Forli. "Autodock vina 1.2.0: New docking methods, expanded force field, and python bindings." *J Chem Inf Model* 61 (**2021**): 3891-98. 10.1021/acs.jcim.1c00203. https://www.ncbi.nlm.nih.gov/pubmed/34278794.

28. Trott, O. and A. J. Olson. "Autodock vina: Improving the speed and accuracy of docking with a new scoring function, efficient optimization, and multithreading." *J Comput Chem* 31 (**2010**): 455-61. 10.1002/jcc.21334. https://www.ncbi.nlm.nih.gov/pubmed/19499576.

29. Aulifa, D. L., S. R. Amirah, D. Rahayu, S. Megantara and M. Muchtaridi. "Pharmacophore modeling and binding affinity of secondary metabolites from angelica keiskei to hmg co-a reductase." *Molecules* 29 (**2024**): 2983. 10.3390/molecules29132983. https://www.ncbi.nlm.nih.gov/pubmed/38998937.

30. Kuzmic, P. "A steady state mathematical model for stepwise "slow-binding" reversible enzyme inhibition." *Anal Biochem* 380 (**2008**): 5-12. 10.1016/j.ab.2007.11.027. https://www.ncbi.nlm.nih.gov/pubmed/18206642.

31. Amine, A., L. El Harrad, F. Arduini, D. Moscone and G. Palleschi. "Analytical aspects of enzyme reversible inhibition." *Talanta* 118 (**2014**): 368-74. 10.1016/j.talanta.2013.10.025. https://www.ncbi.nlm.nih.gov/pubmed/24274310.

32. Zaru, R., S. Orchard and C. UniProt. "Uniprot tools: Blast, align, peptide search, and id mapping." *Curr Protoc* 3 (**2023**): e697. 10.1002/cpz1.697. https://www.ncbi.nlm.nih.gov/pubmed/36943033.
